# Pleiotropic Mechanisms Indicated for Sex Differences in Autism

**DOI:** 10.1371/journal.pgen.1006425

**Published:** 2016-11-15

**Authors:** Ileena Mitra, Kathryn Tsang, Christine Ladd-Acosta, Lisa A. Croen, Kimberly A. Aldinger, Robert L. Hendren, Michela Traglia, Alinoë Lavillaureix, Noah Zaitlen, Michael C. Oldham, Pat Levitt, Stanley Nelson, David G. Amaral, Irva Herz-Picciotto, M. Daniele Fallin, Lauren A. Weiss

**Affiliations:** 1 Department of Psychiatry and Institute for Human Genetics, University of California, San Francisco, California, United States of America; 2 Department of Epidemiology, Johns Hopkins Bloomberg School of Public Health, Baltimore, Maryland, United States of America; 3 Division of Research, Kaiser Permanente Northern California, California, United States of America; 4 Center for Integrative Brain Research, Seattle Children's Research Institute, Seattle, Washington, United States of America; 5 Université Paris Descartes, Sorbonne Paris Cité, Faculty of Medicine, France; 6 Department of Medicine, University of California, San Francisco, San Francisco, California, United States of America; 7 Department of Neurological Surgery, University of California, San Francisco, San Francisco, California, United States of America; 8 Program in Developmental Neurogenetics, Institute for the Developing Mind, Children’s Hospital Los Angeles and Department of Pediatrics, Keck School of Medicine, University of Southern California, Los Angeles, California, United States of America; 9 Department of Human Genetics, David Geffen School of Medicine, University of California, Los Angeles, Los Angeles, California, United States of America; 10 Department of Psychiatry and Behavioral Sciences, Medicine and Medical Investigation of Neurodevelopmental Disorders (M.I.N.D.) Institute, University of California, Davis School of Medicine, Sacramento, California, United States of America; 11 Department of Public Health Sciences and Medicine and Medical Investigation of Neurodevelopmental Disorders (M.I.N.D.) Institute, University of California, Davis School of Medicine, Sacramento, California, United States of America; 12 Department of Mental Health, Johns Hopkins Bloomberg School of Public Health, Baltimore, Maryland, United States of America; The Wellcome Trust Centre for Human Genetics, University of Oxford, UNITED KINGDOM

## Abstract

Sexual dimorphism in common disease is pervasive, including a dramatic male preponderance in autism spectrum disorders (ASDs). Potential genetic explanations include a liability threshold model requiring increased polymorphism risk in females, sex-limited X-chromosome contribution, gene-environment interaction driven by differences in hormonal milieu, risk influenced by genes sex-differentially expressed in early brain development, or contribution from general mechanisms of sexual dimorphism shared with secondary sex characteristics. Utilizing a large single nucleotide polymorphism (SNP) dataset, we identify distinct sex-specific genome-wide significant loci. We investigate genetic hypotheses and find no evidence for increased genetic risk load in females, but evidence for sex heterogeneity on the X chromosome, and contribution of sex-heterogeneous SNPs for anthropometric traits to ASD risk. Thus, our results support pleiotropy between secondary sex characteristic determination and ASDs, providing a biological basis for sex differences in ASDs and implicating non brain-limited mechanisms.

## Author Summary

Autism Spectrum Disorders (ASDs) make up a debilitating neurodevelopmental disorder class. It has been known for a long time that more males than females are affected, but despite much speculation there is no clear etiological reason for this sex bias. As ASDs are highly heritable, we examined evidence in single nucleotide polymorphism (SNP) data for five plausible genetic models that could generate sex bias. We identified distinct genome-wide significant loci in each sex-specific dataset, and evaluated support in five analyses: 1) In contrast to rare variant contribution, we find no evidence for increased SNP genetic load in females. 2) Sex-heterogeneity is demonstrated on the X-chromosome. 3) We uncover no evidence for hormone-responsive genes being overrepresented in association signals. 4) We identify no signature for genes differentially brain-expressed between males and females contributing to ASDs. 5) We observe a strong signal of excess association in the same regions of the genome showing sex-heterogeneity in anthropometric traits. This latter finding is striking, implicating general sexual dimorphism as opposed to brain- or behavior-specific origins for sex differences contributing to ASDs.

## Introduction

Autism spectrum disorders (ASDs) are characterized by deficits in use of language and social communication, sensory challenges, restricted interests, and repetitive behaviors that manifest in the first years of life. ASDs are estimated to occur in 1/42 boys and 1/189 girls, and are among the most heritable common disorders[1]. Estimates of heritability for idiopathic ASDs range between 38% and 90%, and autism-related traits in the general population are similarly heritable[2–9]. An emerging body of evidence has identified a wide array of potential non-genetic risk factors[10,11]. Nevertheless, the biological underpinnings and relevant environmental risk factors for ASDs are mostly unknown; thus, the nearly five-fold difference in prevalence between males and females may provide critical clues. Sexual dimorphism is extensive, begins early in development, and can be mediated primarily by hormonal or genetic (46, XX vs. 46, XY) differences or by interaction between the two. In humans, hormonal and genetic factors are difficult to dissociate and often do not correspond to animal models. Despite much speculation, there is no definitive evidence regarding why males are more susceptible to ASDs[12].

Several testable genetic models could explain the reduced risk observed in females for idiopathic ASDs. 1) A multifactorial liability threshold model for genetic risk loci, whereby the same alleles affect males and females equally, but females have a higher threshold (for biological or societal reasons) requiring more polygenic load or stronger highly penetrant mutations to be affected or diagnosed due to the modifying effects of sex; 2) Specific susceptibility factors encoded on the X or Y chromosome that affect males, but not females, due to lack of Y or compensatory second copy of X; 3) Specific autosomal risk factors with different effects in males and females due to hormonally-mediated or otherwise-mediated sexual dimorphism, i.e. ‘autism’ is to some degree a different biological disorder in males and females due to gene-sex interaction; 4) A major influence of androgen levels[13]. If these effects are mediated via genes responsive to steroid hormones in their expression, we can hypothesize a role for steroid-responsive genes in genetic liability to ASDs; 5) Pleiotropy with general mechanisms of sexual dimorphism. Since variation in secondary sex characteristics (i.e. height, weight, hip, and waist circumference) is strongly heritable, this model would lead to the same genetic programs showing sex-heterogeneous signals for anthropometric traits exhibiting disproportionate contribution to ASD association. These distinct models are not mutually exclusive, thus in the present report we investigated evidence that would support each of them.

A liability threshold model (1) would dictate that females with an ASD diagnosis would carry more genetic risk than affected males, on average. In support of a liability threshold model, previous studies show that females with ASDs are often more severely affected, with lower IQ and more frequent co-morbidities such as epilepsy[14–16]. Similarly, the difference in prevalence between males and females is lowest for the most severely affected individuals and highest for those who are highest-functioning on the spectrum[17]. Severe features could indicate a greater burden of modest inherited risk factors (as tested in this study), more highly penetrant risk factors likely to be non-inherited, or both. (Note that these predictions about risk factors are true regardless of whether affected females comprise a fair representation of ASD traits in the population or undergo diagnostic bias resulting in recognition of only the more severe cases; the liability threshold represents the empirical one for obtaining an ASD diagnosis.) In support of some features of the liability threshold model, many highly penetrant ASD causes, such as *de novo* deletions, seem to have closer to equal sex ratios[18–20]. Recent exome sequencing studies have found that female cases have a greater proportion of *de novo* loss-of-function mutations and that single nucleotide variants (SNVs) identified in female cases exhibit an excess of deleterious predictions[21–24]. A model whereby females require higher inherited genetic loading to be affected than males would suggest that females should have an increased burden of family history. This model has some suggestive support in recent studies[25,26], but lack of evidence in other studies[27]. Thus, it could be feasible that although individually strong risk factors like *de novo* mutations are enriched in females with ASDs, modest polygenic influence of common polymorphisms could contribute proportionately more or solely to male risk if they are generally insufficient to achieve the higher female threshold. Thus, there are two opposite but equally plausible models that can be simultaneously evaluated: 1) The majority of both male and female ASD is heritable (not captured in *de novo* mutations); strong sex bias is present in those likely to have risk from SNPs; therefore, the female liability threshold only considering SNPs may be increased compared to males (it requires a higher SNP burden for a female to be affected). 2) For ASD overall, the observation of increased *de novo* mutation in females and more severe ID in females with ASD may imply that SNP risk is not sufficient for a female to be affected; therefore, considering only SNPs, female genetic burden may appear decreased compared with males (based on SNPs, ASD would appear not to be heritable).

Although rare genetic events causing ASDs have been identified on the X chromosome, such as mutations in the *NLGN3*, *NLGN4X*, *ARX*, *MECP2*, *FMR1* genes, microdeletion, and aneuploidy[28], there is little evidence that common risk factors of strong effect for ASDs lie on the X or Y chromosomes to support the sex chromosome risk model (2)[29–31]. A recent study of single nucleotide polymorphism (SNP)-based heritability estimated a disproportionately low contribution of the X chromosome to polygenic risk based on its length[32]. An exome sequencing study has estimated that 1.7% of male ASDs may be comprised of individuals with rare X-linked loss-of-function SNVs[33]. With respect to model 3, male-specific autosomal linkage consistent with autosomal gene-sex interaction (3) has been identified, including a replicated region of chromosome 17[34–36]. In addition, autosomal dominant single-gene RASopathy syndromes have gene-sex interaction with NF1 showing male bias in ASD symptoms and Noonan syndrome showing a lack of sex bias[37]. However, gene-sex interaction has not been investigated in modern genome-wide association study (GWAS) datasets. Theories for excess male hormones characterizing ASDs (4) have led to investigation of testosterone levels in ASDs, with varied results[38–41]. A recent study found evidence for increased levels of steroid hormones in the amniotic fluid samples of subjects who went on to develop an ASD[42]. However, the androgen theory of ASDs has not yet been comprehensively investigated at the genetic level. To our knowledge, no one has studied the relationship of secondary sex characteristics and behavioral sexual dimorphism (5).

Here, we investigated five genetic models of sexual dimorphism in ASDs: 1) We examined evidence for a higher common polymorphism genetic load in the lower-prevalence sex. 2) We investigated sex-heterogeneity and association enrichment specific to the X chromosome. 3) We assessed the contribution of g x sex interaction across the autosomes. 4) We evaluated the role for genes whose expression is influenced by steroid hormones or sexually-dimorphic in the brain. 5) Finally, we estimated whether SNPs exhibiting sex-heterogeneous association with anthropometric traits contribute to ASD risk implicating pleiotropy with secondary sex characteristics.

## Results

### Sex-specific Association Analysis

**Fig 1:**
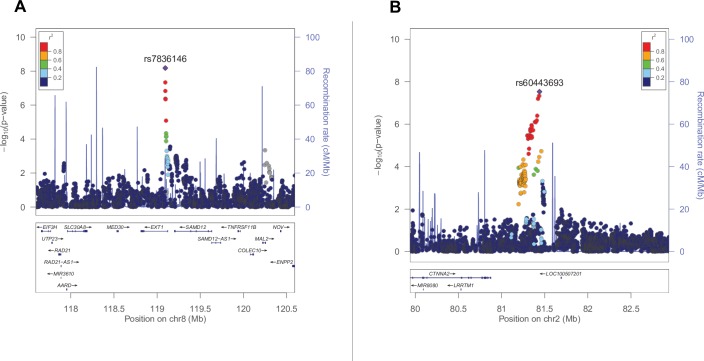
Plot of region surrounding most significant SNPs. (A) Male ASD association results surrounding rs7836146 in the region chromosome 8: 117.6–120.5 Mbp. (B) Female ASD association results surrounding rs60443693 in the region chromosome 2: 79.9–82.9 Mbp. Plots were generated using LocusZoom[88] (see URLs). SNP position information based on hg19 reference version and LD and recombination rate data based on 1000 Genomes (November 2014) EUR population. SNPs are colored based on linkage disequilibrium (LD) correlation (r^2^), or colored gray if no LD information exists. The overlaid blue line corresponds to the recombination rate.

In order to test the different hypotheses of sex-specific genetic architecture, we obtained the largest sex-specific datasets currently feasible. Recent analyses support the strategy of combining datasets with different ascertainment or diagnostic criteria to maximize power; increased sample size appears to have much greater impact than decreased homogeneity[43,44]. In order to achieve maximal sample size, we utilized previously published GWAS data [Autism Genetic Resource Exchange (AGRE)-Weiss[45], AGRE-Wang[46], Autism Genome Project (AGP)[47], Early Markers for Autism (EMA)[48], SSC[43]; N = 6,567 trios (16% female), N = 625 cases (21% female), and N = 377 controls (19% female)]. To these data, we added samples we genotyped at University of California San Francisco (UCSF) and/or via collaboration with a number of other consortia [UCSF, Childhood Autism Risks from Genetics and the Environment (CHARGE)[49], Study to Explore Early Development (SEED)[50], Autism Phenome Project (APP), Tummy Troubles (TT)[51,52], Interactive Autism Network (IAN)[53]; N = 195 trios (44% female), N = 1,259 cases (16% female), and N = 1,127 controls (37% female)] (Table 1, see URLs). Within each genotyping technical batch (Table 2), quality control was performed, and each dataset was imputed to the 1000G reference panel, with an additional round of quality control for imputed data (Materials and Methods). All imputed datasets were then merged, and SNPs present in 90% of the total dataset were retained. From this mega-dataset, we extracted all complete trios (N = 6,762) for transmission disequilibrium test (TDT) analysis and utilized the remaining data (N = 3,388) in a case-control (CC) analysis. Finally, we performed a meta-analysis of the TDT and CC results for the complete combined-sex dataset (N = 10,150), as well as the male-specific (trios with male probands and male cases vs. male controls, N = 8,207) and female-specific (trios with female probands and female cases vs. female controls, N = 1,943) datasets. One SNP met genome-wide significance (*P* = 5 x 10^−8^) in the combined-sex dataset (rs7836146 near *EXT1*) (Table 3, S1 Fig). Two SNPs in one locus met genome-wide significance in the male-specific dataset (rs7836146 and rs7835763 near *EXT1*) (Table 3, Fig 1A). Notably, patients with rare mutations in *EXT1* have been previously described to have ASDs[54]. Three SNPs in one locus reached genome-wide significance in the female-specific dataset (rs60443693, rs12614637, and rs140431641 in between *CTNNA2* and *SUCLG1*) (Table 3, Fig 1B). Of the top association SNPs (*P* < 10^−6^), each of the independent loci in females show strong sex-heterogeneity (Cochran’s Q, *P* < 10^−3^) and two of the five male independent loci (both on the X chromosome) show sex-heterogeneity (*P* < 0.05). In the combined-sex association results, one locus additionally shows sex-heterogeneity (*P* < 0.05) (S1 Table). None of the top sex-specific associations show within-sex differences comparing high vs. low IQ groups, suggesting the sex-specificity is not confounded by ASD severity differences (S1 Table).

**Table 1:** ASD Datasets.

Dataset	Number of trios (% Females)	Number of cases (% Females)	Number of controls (% Females)	% Caucasian
**AGRE-Wang**[46]	1,641 (22)	148 (34)	0	75
**AGRE-Weiss**[45]	372 (18)	3 (0)	0	87
**Autism Genome Project (AGP)**[47]	2,459 (13)	40 (18)	0	86
**Autism Phenome Project (APP)**	0	141 (11)	79 (37)	52
**Childhood Autism Risks from Genetics and the Environment (CHARGE)**[49]	112 (52)	333 (6)	296 (16)	49
**Early Markers for Autism (EMA)**[48]	0	421 (17)	377 (19)	34
**Interactive Autism Network (IAN)**[89]	0	109 (19)	0	72
**Simons Simplex Collection (SSC)**[43]	2,095 (13)	13 (23)	0	76
**Study to Explore Early Development (SEED)**[50]	0	585 (20)	719 (46)	55
**Tummy Troubles (TT)**[51,52]	0	43 (53)	33 (48)	83
**UCSF/Hendren**[69,70]	0	22 (9)	0	23
**UCSF/Weiss**	83 (33)	26 (27)	0	40

The table describes the following information about each dataset used in our analysis: final number of complete trio sets (unaffected mother and father, child with an ASD), final number of individuals with ASD, and final number of unrelated individuals without ASDs. Proportion female is given for each dataset, and proportion Caucasian, as determined by visual inspection of MDS plots.

**Table 2:** Quality control measures for each ASD dataset.

Technical set	Dataset	Genotyping platform	HWE	ME	MAF	Missing rate
**1**	AGP[47]	Illumina Infinium 1Mv1 array	1x10^-10^	10	0.01	0.02
**2**	AGRE-Wang[46]	Illumina HumanHap550 BeadChip	1x10^-4^	10	0.01	0.06
**3**	AGRE-Weiss[45]	Affymetrix 5.0 SNP array	1x10^-10^	10	0.01	0.03
**4**	EMA[48]	Affymetrix Axiom EUR array	1x10^-10^	10	0.01	0.03
**5**	CHARGE[49]	Affymetrix Axiom EUR array	1x10^-10^	NA	0.01	0.05
**6**	SEED[50]–Johns Hopkins Univ.	Illumina HumanOmni1-Quad BeadChip	1x10^-10^	NA	0.01	0.01
**7**	SSC[43]	Illumina Infinium 1Mv3 (duo) array	1x10^-10^	10	0.01	0.05
**8**	SSC[43]	Illumina Infinium 1Mv1 array	1x10^-10^	10	0.01	0.05
**9**	SSC[43]	Illumina HumanOmni2.5M array	1x10^-10^	10	0.007	0.03
**10**	APP, CHARGE[49], EMA[48], IAN[53], SEED[50]–UCSF, TT[51,52], Hendren[69,70] / Weiss—UCSF	Affymetrix Axiom EUR array	1x10^-10^	10	0.01	0.04

For each technical set, the table lists the datasets included, the genotyping platform, and the following quality filter thresholds used prior to imputation and merging: Hardy-Weinberg equilibrium *P*-value (HWE), number of Mendelian errors (ME), minor allele frequency (MAF), and percent missing data (Missing Rate).

**Table 3:** Top GWAS associations for combined-sex, male-specific and female-specific datasets.

**COMBINED**							
**SNP**	**CHR**	**BP**	**MAF**	**Beta**	**SE**	***P*-value**	**Genes(s)**
rs7836146	8	119095022	0.21	-0.17	0.03	5.6x10^-09^	*EXT1*
rs144955418	X	141650006	0.03	-0.56	0.10	8.1x10^-08^	*MAGEC2 / SPANXN4*
rs117135939	19	53743855	0.06	0.24	0.05	5.6x10^-07^	*ZNF677*
rs6961764	7	133131298	0.50	0.11	0.02	6.1x10^-07^	*EXOC4*
rs113648237	X	5359798	0.04	-0.40	0.08	7.6x10^-07^	*PRKX/NLGN4X*
**MALE**							
**SNP**	**CHR**	**BP**	**MAF**	**Beta**	**SE**	***P*-value**	**Genes(s)**
rs7836146	8	119095022	0.21	-0.18	0.03	6.6x10^-09^	*EXT1*
rs9348610	6	23812225	0.38	-0.14	0.03	1.6x10^-07^	*HDGFL1 / NRSN1*
rs150278852	X	140490159	0.02	-0.82	0.16	2.7x10^-07^	*SPANXC*
rs144955418	X	141650006	0.03	-0.60	0.12	4.1x10^-07^	*MAGEC2 / SPANXN4*
rs145339701	X	126205770	0.03	-0.56	0.11	6.4x10^-07^	*PRR32 / ACTRT1*
**FEMALE**							
**SNP**	**CHR**	**BP**	**MAF**	**Beta**	**SE**	***P*-value**	**Genes(s)**
rs60443693	2	81439635	0.07555	-0.58	0.11	3.0x10^-08^	*CTNNA2 / SUCLG1*
rs7803848	7	133108547	0.3271	-0.29	0.06	2.7x10^-07^	*EXOC4*
rs150388754	8	4037697	0.03857	-0.69	0.14	8.7x10^-07^	*CSMD1*

SNPs with association *P*-value < 10^−6^ are shown, with only the most significant SNP per independent locus shown. dbSNP rsID or position for in/dels are shown (SNP), alongside chromosome, position in base pairs (BP) for hg19, minor allele frequency (MAF) in our dataset, effect size (Beta), standard error (SE), *P*-value, and gene(s) in or nearest to the SNP.

### Genetic Load

**Fig 2:**
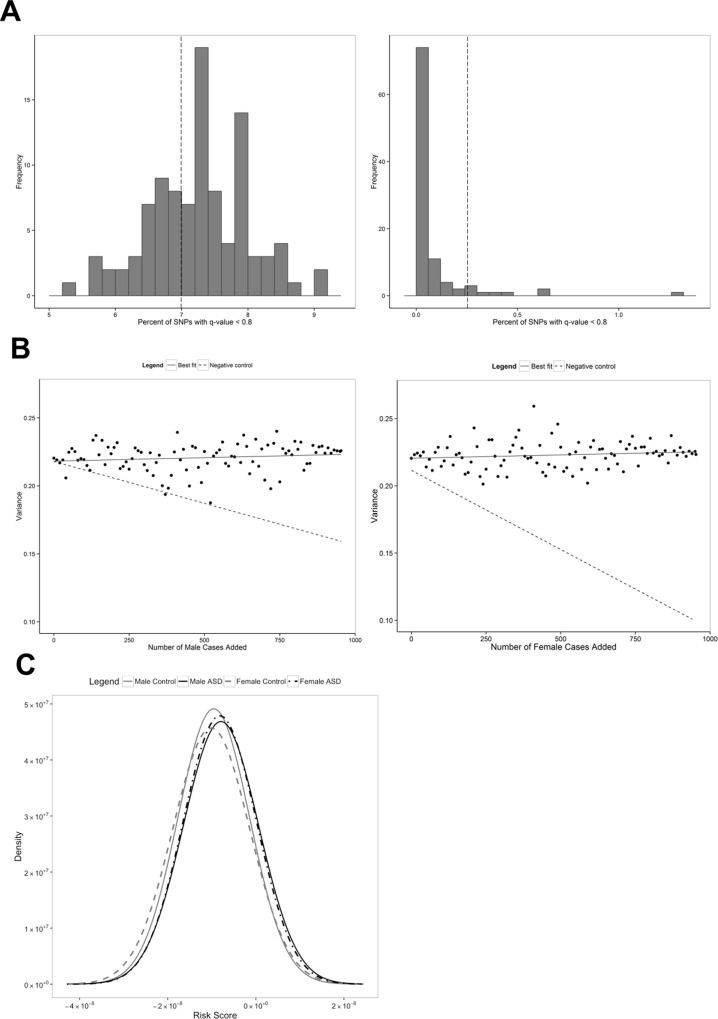
Increased female genetic risk load. (A) Autosomal genetic load. The distributions of sex-permuted autosomal signal enrichment at an FDR q-value threshold of 0.8 are compared to the male-specific and female-specific percent of SNPs with q-value < 0.8 (dashed line indicates males = 6.99%, females = 0.25%). (B) Correlation between the number of male or female cases added and heritability estimate. The solid line displayed is the linear best fit line. The dashed line is the linear best fit line for the correlation between the number of male or female pseudo-controls added and heritability estimate (negative control). (C) Predicted risk scores. Comparison of probability densities of predicted risk scores for males and females with and without ASDs. The order of the distributions from lowest to highest mean risk score (left to right) is: female controls (dashed gray line), male controls (solid gray line), female ASD cases (dashed black line), and male ASD cases (solid black line).

Our first mechanistic hypothesis contributing to sex differences is increased genetic load in the lower-prevalence sex. Although it has been suggested in several studies that rare, highly penetrant risk variants are more strongly enriched in female probands compared to male probands, common polymorphism data has not been examined for sex differences in genetic load. We first assessed potential evidence for enrichment of genetic association signal in female cases (compared to parental genotypes or female controls) versus in male cases (compared with parental genotypes or male controls). In order to adjust for the differential power of these datasets (affected males N = 8,207; affected females N = 1,943), we utilized sex permutations, whereby sex classifications were permuted within technical batch/dataset and study design (trio or case-control) to obtain permuted datasets of mixed sex but equal power to the true male and female datasets (Materials and Methods). We assessed enrichment by setting a false discovery rate (FDR) threshold (q = 0.8) and comparing the proportion of SNPs exceeding this threshold (note that we use FDR only as a metric for comparison, not to assess significance). Male autosomal datasets did not show any enrichment compared to sex-permuted datasets (Fig 1A). However, only 8% of sex-permuted female datasets exceeded the true female autosomal association enrichment (*P* = 0.08) (Fig 2A). This trend could occur due to heterogeneity (e.g. some female-specific association loci not shared by males) or due to increased genetic load in affected females.

Next, we assessed whether SNP-based additive heritability (*h*^*2*^_*g*_) would support a liability-threshold model resulting in increased female genetic load. To do this we utilized our family-based dataset and compared proband genotypes to pseudo-controls (non-transmitted parental alleles). Because the female-specific dataset is underpowered for heritability comparison (Materials and Methods), we set aside the female-specific dataset and a matched male-specific dataset and utilized the remaining independent male-specific dataset for heritability estimation. We then added increasing numbers of female cases in a step-wise manner. For comparison, we did the same with the matched set-aside male-specific dataset. We observed no difference in correlation between the number of female versus male cases included and the observed-scale heritability estimates (Spearman's rank correlation: female rho = 0.195, *P* = 0.06; male rho = 0.195, *P* = 0.06). When using female and male pseudo-controls only, we see a negative correlation between number of male and female pseudo-controls added and observed-scale heritability estimates (Spearman's rank correlation: female rho = -0.951, *P* < 2.2 x 10^−16^; male rho = -0.863 *P* < 2.2 x 10^−16^). These results suggest that on an individual basis females show equivalent SNP-based heritability to males (Fig 2B).

Finally, to assess individual-level risk burden distributions in males and females, we utilized the genome-wide genetic relationship matrix to predict the linear aggregate genetic risk for each individual. In order to carry out sex comparisons, we set aside our complete female datasets and a matched male subset. We used the remaining male data to generate best linear unbiased prediction (BLUP) solutions for each SNP, and applied these to our reserved independent male and female datasets (Materials and Methods). We found that in both males and females, cases showed significantly higher mean SNP risk scores than controls (*P*_*male*_ = 1.82 x 10^−5^; *P*_*female*_ = 6.46 x 10^−7^). However, neither male and female controls nor cases differed significantly from each other in a male-derived risk score (Fig 2C). Similarly, within-sex high vs. low IQ ASD-affected groups did not differ by risk score (S2 Fig).

### X Chromosome

A second plausible genetic mechanism underlying sex differences could be genetic risk encoded on the X chromosome. We identified significant and suggestive association at several loci on the X chromosome, in or between the following genes: *SPANXC*, *PRR32 / ACTRT1*, *PRKX / NLGN4X*, and *MAGEC2 / SPANXN4* (Table 3). Thus, we wanted to test whether the X chromosome is overall enriched in association signal compared with similarly sized chromosomes and whether the X chromosome shows association that is specific to males with ASDs. First, we assessed association enrichment on the non pseudo-autosomal X chromosome utilizing a similar FDR-threshold strategy as above, in comparison with chromosome 7 (similar physical size) and chromosome 17 (similar SNP representation). In females, chromosome X shows equivalent signal to the comparison chromosomes, and in the male-specific dataset, chromosome X shows slightly increased enrichment compared to chromosome 17 (*P* = 0.09) (S2 Table). Performing sex permutations, we observed enrichment in male-specific data compared with sex-permuted data (*P* = 0.04) (S2 Table). This could occur if X-linked loci have stronger effects in males compared to females or sex-limited effects that do not extend to females.

**Fig 3:**
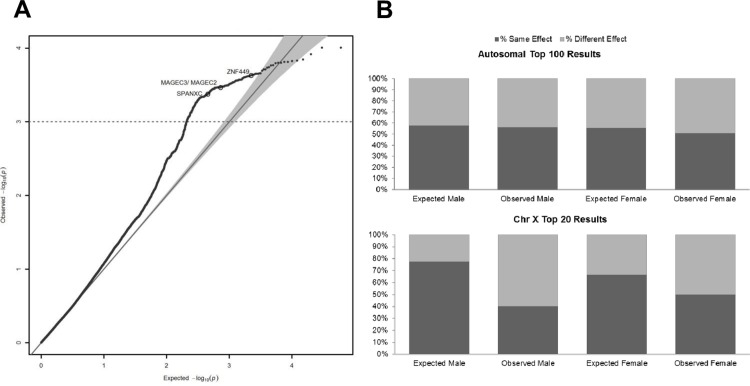
Sex heterogeneity on the X chromosome. (A) Quantile-quantile (QQ) plot of Cochran’s Q results. The QQ plot displays the heterogeneity estimates for SNPs on chromosome X between male-specific and female-specific association results. SNPs with Cochran’s *P* < 10^−3^ and a greater absolute effect size in males are circled. (B) Binomial sign test results. The minor allele direction is compared for the most significant independent SNPs in male-specific or female-specific association results to the opposite sex. 100 autosomal SNPs and 20 chromosome X SNPs were used for comparison. The expected value for each set is based on the mean percent in the same direction from the sex-permuted association results.

We assessed association heterogeneity on the X chromosome via Cochran’s Q statistic. The vast majority of SNPs with heterogeneity *P* < 10^−3^ have larger absolute effect size in females; however, this is attributable to the smaller sample size, as our permuted datasets showed similar results (Fig 3A). We examined 20 independent SNPs that were most significant in the male and female associations on the X-chromosome individually. The true distributions for heterogeneity among top SNPs were compared with sex-permutations as above, but adjusting the FDR level to account for SNPs ascertained for association (FDR_female_ = 0.01; FDR_male_ = 0.2). The male top hits were significant for sex-heterogeneity (*P* < 0.01) and the female top results did not show heterogeneity compared to the sex-permuted association results. As heterogeneity can be due to differing effect sizes in the same direction or no effect (or opposite effect) in one sample, we performed a binomial sign test. Female and male top 20 independent results on the X chromosome were suggestively or significantly depleted of same-direction effects compared with sex-permuted datasets (*P*_*female*_ = 0.06, *P*_*male*_ < 0.01) (Fig 3B).

### Autosomal G x Sex

Third, we wanted to test a hypothesized mechanism of global autosomal gene-by-sex interaction. In contrast to the X chromosome, female and male autosomal top 100 independent SNPs did not show any difference from sex-permuted datasets for direction of effects (Fig 3B). Together, the similar heritability and risk scores for male and female cases and lack of difference in the sign test suggest that much of the autosomal genetic signal is derived from common associations across males and females. In the extreme case of completely different genetic risk determinants, one would expect the heritability and association signal to decrease for both males and females in combined-sex datasets. To verify this prediction, we tested for heterogeneity among the 100 strongest independent autosomal association results for each sex by calculating Cochran’s Q statistic. We then set FDR thresholds adjusted for ascertaining via test statistics (FDR_female_ = 0.001; FDR_male_ = 0.2) and compared to our sex-permuted datasets, which have an even proportion of males and females per permuted set (Materials and Methods). No significant evidence of heterogeneity was observed for top male or female SNP results.

### Gene Set Analyses

Our fourth and fifth hypotheses would result in enrichment of genetic association or sex-heterogeneity concentrated in specific limited sets of genetic variation, such as those likely to differ based on hormonal milieu or those shared with brain-specific or anthropometric secondary sex characteristics. To assess whether specific biological gene sets are likely to show sex differences, we obtained lists of genes with gene expression levels that are 1) androgen-responsive (AR)[55], 2) estrogen-responsive (ER)[56], 3) sexually-dimorphic in early brain development (SD)[57], or 4) sex-correlated (SC) (Materials and Methods). Significance of enrichment was determined by permutation with matched-length genes (Materials and Methods) and significance of sex differences was determined by sex permutation, as above. Lastly, we examined 5) SNPs showing sex heterogeneity in association to anthropometric traits (AH; heterogeneity *P* < 10^−3^ for height, weight, BMI, hip, or waist measurements from the Genetic Investigation of ANthropometric Traits (GIANT) datasets[58]). Significance of enrichment for this set was determined by permutation matched by test statistic in the combined-sex GIANT dataset to control for SNP ascertainment via trait association, and empirical significance for sex differences was calculated by sex permutation (Materials and Methods).

SNPs contained within AR or ER genes experimentally determined to be hormone-responsive did not show enrichment for association signal in either sex or for heterogeneity via sex permutation. Nor did SD genes with sexually-dimorphic expression level or SC genes whose fetal brain expression is correlated or anti-correlated with ‘male-ness’ (e.g. Y-encoded gene expression) show excess association or sex differences.

In order to test whether the same genetic influences resulting in sexually-dimorphic anthropometric traits are enriched in sexually-dimorphic behavioral disorders like ASDs, we tested whether SNPs showing sex heterogeneity of association signal to anthropometric traits (AH) were enriched for association signal or sex differences in ASDs. Indeed, in females with ASDs, AH SNPs showed enrichment for association signal compared to SNPs equally associated with anthropometric traits but not ascertained for sexual-dimorphism (*P*_*female*_ < 0.01) (Table 4). To determine if this result is specific to females, we assessed enrichment of AH SNPs in the ASD combined-sex dataset and observed similar results (*P*_*all*_ < 0.01). We wanted to determine whether this observation might be exclusive to ASD, so we obtained summary statistics for two equivalently imputed datasets from the psychiatric genomics consortium (PGC), schizophrenia (SCZ)[59] and bipolar disorder (BIP)[60]. Although neither disorder has overall sex prevalence differences, we observed similar AH SNP enrichment for BIP (*P*_*all*_ < 0.01), but not for the well-powered SCZ dataset. Previous work has revealed sexual dimorphism in onset, course, and co-morbidities of BIP[61] and in animal models, and suggested that it may be driven by endocrine systems[62]; thus the AH SNP enrichment in ASD is not unique but is also unlikely to be an artifact manifest in all GWAS data.

**Table 4:** Anthropometric-Heterogeneous SNPs.

SNP	CHR	Base Position	Gene(s)	Male—ASD	Female—ASD	AH trait	AH sex difference	AH sex
**rs6717858**	2	165539661	*COBLL1*	0.73 (-)	2.2x10^-3^ (-)	WCadjBMI	6.4x10^-6^	F+
**rs6063796**	20	51093873	*ZFP64 / TSHZ2*	5.1x10^-3^ (+)	0.97 (-)	BMI	7.2x10^-6^	F+
**rs9893250**	17	49901007	*CA10*	0.22 (-)	5.4x10^-3^ (+)	WCadjBMI	1.9x10^-4^	F-
**rs1871637**	10	61586443	*CCDC6*	5.0x10^-3^ (+)	0.027 (+)	WCadjBMI	2.1x10^-4^	F+
**rs17241417**	13	20282946	*PSPC1*	3.6x10^-3^ (-)	0.26 (-)	WHRadjBMI	2.4x10^-4^	M-
**rs6989759**	8	49296878	*UBE2V2 / EFCAB1*	0.60 (-)	6.3x10^-3^ (-)	HIPadjBMI	2.4x10^-4^	M-
**rs425930**	6	5108419	*LYRM4*	7.9x10^-3^ (+)	0.17 (+)	HIPadjBMI	2.7x10^-4^	M-
**rs6841228**	4	147071801	*ZNF827 / LSM6*	6.1x10^-3^ (-)	0.024 (+)	WCadjBMI	3.0x10^-4^	M-
**rs10815468**	9	6784726	*KDM4C*	5.4x10^-2^ (-)	3.3x10^-3^ (-)	WCadjBMI	3.1x10^-4^	M-
**rs10105804**	8	94390503	*TRIQK / FAM92A1*	0.65 (-)	6.2x10^-3^ (+)	WHRadjBMI	3.7x10^-4^	M-
**rs755647**	10	7162204	*PRKCQ / SFMBT2*	3.5x10^-3^ (-)	0.35 (-)	HIPadjBMI	3.8x10^-4^	M+
**rs4814605**	20	17327709	*PCSK2*	4.1x10^-3^ (+)	0.18 (+)	HIPadjBMI	4.1x10^-4^	M-
**rs12892860**	14	78949321	*NRXN3*	0.80 (-)	4.0x10^-3^ (+)	WHRadjBMI	5.3x10^-4^	M-
**rs3105153**	5	54411238	*CDC20B*	0.67 (+)	6.2x10^-3^ (-)	WHRadjBMI	5.3x10^-4^	M-
**rs10501068**	11	26769636	*SLC5A12 / ANO3*	0.20 (+)	9.5x10^-3^ (-)	Height	5.4x10^-4^	F-
**rs6761469**	2	58581018	*FANCL / BCL11A*	9.9x10^-3^ (+)	0.61 (+)	BMI	5.6x10^-4^	F-
**rs4673823**	2	215249581	*SPAG16*	9.2x10^-3^ (+)	0.21 (-)	WHRadjBMI	6.2x10^-4^	M+
**rs9866112**	3	116974445	*LSAMP / IGSF11*	0.37 (+)	3.1x10^-3^ (+)	HIPadjBMI	6.3x10^-4^	F-
**rs12417170**	11	17460084	*ABCC8*	0.19 (-)	3.8x10^-3^ (-)	WHRadjBMI	7.9x10^-4^	M+ / F-
**rs7386698**	8	17565118	*MTUS1*	3.8x10^-3^ (+)	NA	WCadjBMI	8.4x10^-4^	M-
**rs7179963**	15	71643803	*THSD4*	5.3x10^-3^ (-)	0.061 (-)	BMI	8.8x10^-4^	F+
**rs6138457**	20	24973506	*APMAP*	0.44 (-)	9.3x10^-3^ (-)	WCadjBMI	8.8x10^-4^	F+
**rs7731395**	5	102361761	*PAM*	0.84 (+)	9.6x10^-3^ (-)	BMI	9.0x10^-4^	M-
**rs3786803**	19	30963604	*ZNF536*	7.8x10^-3^ (-)	0.45 (+)	HIPadjBMI	9.1x10^-4^	F-
**rs11113753**	12	108524734	*WSCD2*	8.4x10^-3^ (-)	0.58 (+)	WCadjBMI	9.7x10^-4^	M-

The tables list the anthropometric-heterogeneous (AH) SNPs with male-specific and female-specific ASD association results *P* < 0.01, with only the most significant SNP per independent locus shown. The information listed includes dbSNP rsID (SNP), chromosome (CHR), position in base pairs (BP) for hg19, gene(s) in or nearest to the SNP, *P*-value (minor allele effect direction) of SNP in sex-specific ASD analyses (Male–ASD and Female–ASD), Cochran’s Q *P*-value (AH Sex Difference) indicating sex heterogeneity in the respective anthropometric trait (AH Trait), and anthropometric trait male or female association (M / F) and minor allele effect direction (+ / -) for results *P* < 0.05 (AH Sex).

In order to understand the functional characteristics of the AH SNPs, we tested for overlap with our gene sets, and found that they showed significant overlap with AR and ER datasets compared with permuted SNP lists (*P* < 0.01, each), although the amount of overlap was small. We also found suggestive overlap with predicted binding sites for hormone-responsive transcription factors *AR*, *ESR1* and *LEF1* (*P* < 0.1, each), but not for *ESRRA*, *ESRRB*, or *NKX3-1*[63]. We did not find disproportional overlap with SD or SC genes in the developing brain.

## Discussion

In this study, we set out to assess sex-specific mechanisms in common polymorphism association signal for ASDs. We gathered the largest feasible dataset to do so, however the strength of our conclusions are limited by the sample size we achieved and diverse study designs of component datasets, including mixed ancestry and ethnicity. We mitigated the impact of study differences on our sex-specific conclusions to the degree possible by permuting within technical batches/datasets and study designs to exclude some foreseeable sources of confounding, but our overall study may be reduced in power by the heterogeneity present. On the other hand, our use of diverse-ancestry datasets may render our results applicable to a broader group of populations. Our primarily family-based datasets may similarly contribute to relatively robust results due to perfect genetic matching between parents and offspring, but at the same time features such as assortative mating may reduce overall power compared with using population-based controls[32]. In addition, multiple analyses were performed in order to assess five potential hypotheses, calling for replication of each individual finding in the future when sufficient datasets become available. Despite these limitations, we describe results below providing evidence (or absence thereof) for genetic mechanisms of 1) increased genetic load in the lower-prevalence sex, 2) disproportionate ASD risk contained on the X chromosome, 3) global autosomal gene-by-sex interaction, 4) hormone-driven genetic sex differences, and 5) general pleiotropy resulting in shared mechanisms between ASD risk and secondary sex characteristics.

Our first hypothesis was a major difference in genetic load between males and females. We showed similar heritability estimates on the observed-scale when females were included, and similar male-derived risk prediction scores in female and male cases. However, we observed a trend towards enrichment of association signal in female-only analysis compared with permuted-sex analyses. Thus, we do not see a decrease in common polygenic load in affected females, which might have been consistent with a disproportionate or nearly exclusive role for rare or *de novo* genetic variants contributing to female ASDs and associated with the more severe phenotypic manifestations. On the other hand, we do not observe a striking excess of genetic load or enrichment in females as has been demonstrated for *de novo* loss of function variants in overlapping datasets[18–24]. We do not see differences in genetic load comparing within-sex low vs. high IQ groups. Despite our limited power to detect modest relative differences, we do observe an extremely clear case-pseudocontrol difference, demonstrating our power to detect large differences even in a mixed-ancestry dataset. Together these results suggest that each component of genetic architecture (rare variants, common polymorphisms, inherited, *de novo*, etc.) should be considered separately for sex differences in ASDs, and potentially in other disorders.

Our second hypothesis was a major role for X chromosome polymorphisms. Male-specific analyses revealed sex-heterogeneity specific to the X chromosome and several X-linked loci associated at genome-wide significant levels. In addition, we find association in the combined-sex dataset with polymorphism in *NLGN4X*, previously reported as having rare inherited variants associated with ASDs in males[64,65]. Female X chromosome associations also show suggestive results in the sign test, indicating that there may be female-acting and female-specific risk loci on the X chromosome. These results, taken together, suggest a role for common polymorphisms on the X chromosome in addition to the more well-described role for rare X-linked loci or Mendelian diseases in ASD[28](33). Although many complex trait studies exclude the X chromosome when examining genome-wide autosomal SNPs, our results indicate additional analysis of SNPs on the X chromosome in sex-specific datasets may be worthwhile.

Third, we proposed global autosomal sex-heterogeneity. Despite the relatively smaller sample size, we identify a genome-wide significant association signal in females when analyzed alone, which shows strong sex-heterogeneity (*P* < 10^−8^), but no difference in low vs. high IQ groups (S1 Table). This locus is near the *CTNNA2* locus encoding alpha-2 catenin, a key neurodevelopmental gene. In addition, one of the loci identified in the combined-sex association results at *P* < 10^−6^, near *EXOC4*, showed significantly stronger effect in female cases. This locus, as well as male-identified *EXT1* and *NRSN1* loci in our ASD data, have recently been implicated in population-based learning and memory GWAS[66,67]. Our results thus suggest that potential sex differences should be investigated in these cognitive phenotypes. We did not find evidence for global sex-heterogeneity in association or heritability for the autosomal genome; nor did we identify a locus on chromosome 17 that might explain previous sex-specific linkage findings [34–36]. However, our datasets are limited in power to detect subtle effects that might become evident with increased sample sizes and our study design is complicated by combining datasets with different ascertainment biases.

Fourth, we assessed whether a hormone-driven mechanism might be evident in genetic set enrichment. We were unable to identify genetic support for an androgen-driven mechanism for ASD risk loci, represented by genes with expression levels influenced by androgens. Nor did we find evidence for markedly increased influence of genes with sexually-dimorphic brain expression in early development. As these represent small and imperfectly-selected sets of SNPs to represent functional categories, our power may be limited to conclude a lack of effect from these mechanisms. In addition, it is possible that dimorphic gene expression may arise through sex-related differential environmental effects but not show association with genetic variation in these genes.

Our final hypothesis was substantial pleiotropy between anthropometric and complex disease sex differences. We found strong evidence that variants with sexually-dimorphic effects on anthropometric traits contribute disproportionately to ASD association. Our interpretation of this result is that the same mechanisms acting on secondary sex characteristic differences later in life may influence ASD risk in early development. As these loci were identified via anthropometric traits such as height, weight, and waist/hip measurements, our finding suggests general pleiotropy rather than brain-limited or behavioral-specific influences on sex-specific ASD risk. Although we obtained similar results in bipolar disorder, independent replication of these results in additional ASD datasets would be ideal.

There has been much discussion of potential diagnostic bias towards males influencing the observed prevalence differences by sex[68]. However, the disproportionate enrichment (particularly in affected females) of anthropomorphic-heterogeneous SNPs indicates that general biological mechanisms related to sexual dimorphism contribute prominently to ASD risk and could be investigated in other sex-biased behavioral and developmental disorders. Further work may clarify the functions of this set of SNPs and the means by which they act on ASD risk, and could help to quantify limitations on the effects of diagnostic bias in observed prevalence differences.

Overall, our study complements recent identification of rare variants in ASD-affected females by assessing polygenic common SNP association in a sex-specific framework[24]. We report comprehensive evidence of common polymorphic X-linked loci contributing to ASD risk and sex-heterogeneity specific to X-linked loci. Notably, our data highlight the importance of general mechanisms of sexual dimorphism in the etiology of ASDs, and future research may be able to clarify specific biological mechanisms involved and to what degree our findings here may apply to other sex-biased disorders.

## Material and Methods

### Datasets

Information about diagnosis and inclusion/exclusion criteria for each dataset is described in Supplemental Note 1. Genotype data for each dataset are summarized in Table 1. Previously published GWAS data included Autism Genetic Resource Exchange (AGRE)-Weiss[45], AGRE- Wang[46], Simons Simplex Collection (SSC)[43], Autism Genome Project (AGP)[47], and Early Markers for Autism (EMA)[48]. We obtained data by application to AGRE, SSC, dbGAP (AGP), or as study investigators (EMA) (see URLs). Genotype data and phenotype data were utilized as provided, with additional quality control steps described below. Normalized intelligence quotient (IQ) or developmental quotient (DQ) data indicating low (<70) or high (>80) functioning categories were available for 3,571 affected males (2,017 low, 1,554 high) and 619 affected females (405 low, 214 high) and used for secondary analyses (see S1 Note).

Genotyping was performed at the University of California San Francisco (UCSF) genomics core facility for unpublished trios and case-control samples from UCSF/Weiss, UCSF/Hendren[69,70], Tummy Troubles (TT)[51,52], Interactive Autism Network (IAN)[53], Childhood Autism Risks from Genetics and the Environment (CHARGE)[49], Autism Phenome Project (APP), and for a portion of the multisite Study to Explore Early Development (SEED)[50] study (see URLs). Affymetrix Axiom EUR arrays were used, according to manufacturer protocols[71]. Additional unpublished data from the SEED cohort were genotyped on the Illumina Omni1M Quad BeadChip at the Johns Hopkins SNP Center, according to manufacturer protocols. For this dataset, quality control measures were applied within technical batches (Table 2), stratified by ancestry. These measures included removal of samples with a call rate less than 98%, a sex discrepancy, relatedness (PI-HAT > 0.2), or excess hetero- or homozygosity. [Note that previous studies have shown inflated PI-HAT estimates in multi-ethnic datasets, thus our relatively high PI-HAT threshold is appropriate for this study design[72]. Additionally, SNPs with a missing call rate greater than 1%, monomorphic, with minor allele frequency (MAF) less than 1%, or which deviated significantly (*P* < 1.0x10^-10^) from Hardy Weinberg Equilibrium (HWE) were removed. All datasets were anonymized and patient identifiers, except for affection status and sex, were removed in the genotyping datasets used by the investigators.

Saliva and blood samples collected for patients recruited specifically for this study for the dataset UCSF/Weiss were approved for research use by UCSF Committee on Human Research (IRB #: 10–02794). We obtained informed consent and HIPAA authorization for all participants. We have made these data available on The National Database for Autism Research (NDAR) (see Accessions, see URLs). According to the following criteria set by the UCSF Committee on Human Research (1) coded private information or specimens not collected specifically for the current research project, and for which (2) by agreement or by IRB-approved written policies the key to coded human subjects data will not be released to investigators analyzing the data, the other datasets utilized for this study were considered as non-human subject data by the UCSF Committee on Human Research.

### Data Preparation

Marker quality was assessed within technical batches; exact thresholds for HWE, call rate, MAF, and Mendel errors for marker exclusion in the different datasets are noted in Table 2. Technical batches were merged within sub-studies to assess individual identity or to check for known and unknown relationships. Relationships indicating confounding family structure were corrected or individuals contributing to confounding relationships were removed. Remaining individuals were assessed for individual call rate, heterozygosity and sex; those who had unresolvable sex (F-het > 0.3–0.35), increased heterozygosity, or genotyping rate < 0.95% were removed. All individual and marker quality control was carried out using PLINK (see URLs)[73,74].

Genotype datasets mapped to hg18 positions were updated to hg19 using the LiftOver tool available from University of California Santa Cruz (UCSC) Genome Browser (see URLs). Post-quality control datasets, separated by genotyping platform, were checked against 1000G phase1v3 reference data using SHAPEIT’s—check function[75,76] (see URLs). Markers that received an error warning had alleles flipped using PLINK’s—flip option; flipped data was rechecked against the reference panel, and finally any markers still receiving an error warning using SHAPEIT’s—check were then excluded from consideration. Refined datasets were then phased utilizing SHAPEIT and 1000G phase1v3 reference data, specifying—duohmm -W 5 to take advantage of pedigree information when available. Phased genotyping datasets were imputed with IMPUTE2 specifying HapMapb37 as the recombination map, 1000G phase1v3 as the reference panel, and an effective population size of 20,000 using the–Ne flag[77] (see URLs). Chromosomes were processed separately in consecutive chunks of 5MB per chunk for imputation. Chunks were concatenated across entire chromosomes and converted back to PLINK binary file format from Oxford gen/sample format for each chromosome separately, keeping only calls with a imputation quality score of >90%. All marker calls were then matched to the reference panel’s marker ID and position to ensure only properly imputed markers remain; any marker presenting an ID and position that were not exact matches to the reference panel were excluded from further consideration. Separated chromosomes were then merged for each dataset. Quality control filters were applied separately for each dataset, eliminating markers with HWE *P*< 1x10^-10^, call rate of 0.95 and greater than 10 Mendel errors where applicable (Table 2). Additionally, SNPs with large differences in MAF between datasets or indication of being flipped between datasets were removed. All datasets were then merged, applying an additional call rate filter of 0.9 and MAF of 0.01 to include only common variants genotyped for the majority of individuals for analysis.

### Association Analysis

Association was assessed in trio-family (unaffected mother and father with ASD affected child) designed studies using the transmission disequilibrium test (TDT) for 6,762 affected probands (1,113 females and 5,649 males). Association was tested in case-control (CC, ASD affected probands and unrelated unaffected controls) datasets using logistic regression considering ten principal components as covariates in order to control for population stratification (S3 Fig). The ten principal components were calculated using PLINK—mds-plot 10—cluster options. No other covariates were used for the analyses. 1,884 cases and 1,504 controls were used for the logistic regression analysis (338 female cases and 492 female controls; 1,546 male cases and 1,012 male controls). Primary association analyses were carried out using PLINK v1.90[73]. The TDT and logistic regression summary statistics were then used as input into METASOFT[78] (see URLs) for a fixed-effects meta-analysis to find combined-sex association results, male-specific association results (trios with male probands and male CC), and female-specific association results (trios with female probands and female CC). The standard GWAS significance threshold of *P* ≤ 5.0x10^-8^ was used to identify genome-wide significant SNPs accounting for approximate independent common variants[79,80].

### Assessment of Sex Specificity by Permutation

In order to test sex-specificity for each analysis relevant to a potential mechanism of sexual dimorphism, sex permutations were performed by randomly permuting sex classifications (i.e. male or female) for each individual. Individuals were permuted within their respective genotype technical batch/dataset and study design (trio or CC) (Table 2) to account for batch effects. The total number of individuals included in each permuted-sex set was matched to the actual number of male or female probands in the batch to account for the difference in power between the sexes (R script available in GitHub). Then, the TDT and logistic regression association tests were performed on the male-permuted and female-permuted (sex-permuted) datasets, and meta-analysis of the TDT and logistic regression summary statistics was implemented.

Association signal was calculated as the percent of SNPs that surpassed a given FDR q-value of 0.8. The FDR threshold was determined by finding the common threshold for all datasets that had a reasonable number of SNPs to utilize for empirical comparison (S3 Table). Note that this FDR is not used to assess significance, only as a metric for comparison. The observed sex-specific association signal was compared to 100 sex-permuted results. The empirical *P*-value for sex specific association was calculated as the proportion of permuted datasets more extreme than the observed data.

### Genetic Load—Heritability Analysis and Risk Prediction

First, pseudo-controls were created based on our trio dataset using PLINK (—tucc) software[74]. A single proband from each family was used, and individuals showing any relatedness (PI_HAT > 0.1) were removed. The final multi-ethnic dataset consisted of 5,311 trio probands and 5,311 pseudo-controls to be used for heritability and risk prediction analysis. Unrelated case-control datasets were excluded from these analyses, as they would be challenging to match precisely by genetic ancestry. Using Genome-wide Complex Trait Analysis (GCTA), we created the genetic relationship matrix (GRM) between all pairs of individuals based on all autosomal SNPs (see URLs) [81]. We calculated the heritability based on the GRM and ten principal component analysis (PCA) eigenvectors as quantitative covariates to account for population stratification[81–83]. Heritability on the observed scale, defined as the genotypic variance divided by the phenotypic variance, was estimated using GCTA program’s unconstrained restricted maximum likelihood (REML) analysis. To assess the effect of female cases on the heritability estimate, we performed the REML analysis in GCTA for differing proportions of added female cases. Starting with a base set of 6,810 male trio probands and pseudo-controls, we added female probands and their matched pseudo-controls in a step-wise manner from 0 to 1,906, the maximum number of females. A total of 97 sets were created, where each additional set contained all the individuals from the previous set plus up to ten pairs. For comparison, we performed a similar step-wise analysis, adding an equal number of male proband and matched pseudo-controls to the base male dataset (R script available in GitHub). As a negative control and to account for sample size, we performed the step-wise heritability analysis with pseudo-controls only. We did this first with female pseudo-controls designated as “cases” and male pseudo-controls designated as “controls”, and then switched female and male pseudo-controls. To avoid technical batch effects, males and females that were added to the base effects were ascertained from the same genotyping technical batch (Table 2). The observed scale heritability estimate was calculated for every set with male or female cases added. Spearman's rank correlation test was conducted to assess significance.

To determine the genetic risk score for individuals, first, we divided the male-specific dataset into a discovery set and a test set. To avoid technical batch effects, we matched the male test set to the number and technical batches of the female set, as done for the heritability analysis. The discovery set contained 6,810 males (3,405 probands and 3,405 pseudo-controls). We predicted the total genetic effect of all SNPs in the male discovery set using best linear unbiased prediction (BLUP) method in GCTA (—reml-pred-rand), and then transformed the solutions for individual autosomal SNPs (—blup-snp)[81–83]. Finally, we predicted the risk score utilizing these SNP-solutions using PLINK (—score) for an independent test male-specific dataset and female-specific dataset (953 probands and 953 pseudo-controls each). To determine the significance of difference in mean predicted risk score between cases and controls and between males and females, we conducted an independent two sample t-test in R (see URLs). Similarly, we assessed mean differences in low IQ (<70) and high IQ (>80) groups within-sex by t-test for individuals with IQ data available overlapping with the independent male and female test datasets (S2 Fig). We also verified that strongly associated SNPs are not the main contributing factor to the difference in the distribution of risk scores between cases and pseudo-controls. This was determined by performing the analysis excluding SNPs with combined-sex ASD association *P* < 1.0x10^-6^.

### X Chromosome Analysis

Sex- specific association signal enrichment was tested for autosomes, chromosome 7, chromosome 17, and the non-pseudoautosomal X chromosome. For the case-control association component, the X chromosome was coded in standard PLINK format where male genotypes are A = 0 and B = 1, and female genotypes are AA = 0, AB = 1, and BB = 2 (see URLs). For mixed-sex analyses (e.g. combined-sex and permuted datasets) sex is also included as a covariate. No changes are required to the TDT for the X chromosome. Male-specific and female-specific association results per chromosome were assessed for enrichment of genetic signal compared to sex-permuted datasets, derived as above (see Assessment of Sex Specificity). In the same manner, association signal for each chromosome was calculated as the percent of SNPs that surpassed the FDR q-value of 0.8.

To assess heterogeneity between males and females on the X chromosome, we calculated the Cochran's Q statistic and *P*-value using METASOFT[78] (see URLs). The Cochran’s Q statistic[84] for each SNP is the weighted sum of squared differences between the effect estimates in the sex-specific analyses and the combined sex meta-analysis. Cochran’s Q follows a chi-square distribution with 1 DF. A significant *P*-value indicates there is a difference in the SNP effect estimates between the male and female specific datasets. We looked at these heterogeneity results in three ways. First, for chromosome X SNPs with a suggestive Cochran’s Q result (*P* < 1.0x10^-3^), we calculated the proportion with a greater absolute effect size, as indicated by the beta from the fixed-effects meta-analysis, in females versus males. We performed the same analysis with the sex-permuted association results to account for power differences in the male and female datasets.

Second, we examined the 20 most significant linkage disequilibrium (LD) independent X chromosome results separately in males and females. We used PLINK–clump option to LD prune the SNPs based on the sex-specific association *P*-values. Separately for males and females, we found the corresponding Cochran’s Q *P*-value for the top 20 SNPs, and calculated the percent of SNPs that surpassed a given FDR q-value of 0.2 in males and 0.01 in females. We determined the FDR q-value based on the value that produced a reasonable percent (between 50–80%) for comparison (S4 Table). We performed the same analysis in the 100 sets of permuted-sex datasets, and compared the observed male-specific and female-specific results to derive an empirical *P*-value. We also verified that individual associated SNPs are not the main contributing factor to heterogeneity by performing the analysis excluding SNPs with male-specific and female-specific ASD association *P* < 1.0x10^-6^.

Lastly, we examined the direction of effect, as indicated by the beta from the meta-analysis, of the LD independent top 20 SNPs for each sex. We conducted a binomial sign test, and compared the results to the 100 permuted-sex results to assess significance (R script available in GitHub).

### Autosomal G x Sex

In order to assess gene-by-sex interaction across the autosomes, we performed genome-wide heterogeneity analysis via Cochran’s Q test, in the same manner as above for the X chromosome. We examined the most significant 100 independent autosomal results for males and females, which were filtered using PLINK—clump. In the same method as described above, for these top 100 SNPs in the sex-specific association results, we calculated the proportion that had a Cochran’s Q result above or equal to an FDR q-value of 0.2 in males and q-value of 0.001 in female autosomes. The FDR q-value threshold was chosen separately in males and females to avoid saturation of results and allow for reasonable comparison (S4 Table). Using the same method, we calculated heterogeneity levels of the sex-permuted association results to derive an empirical distribution for comparison. We compared our true sex-specific heterogeneity enrichment values to the empirical distribution to calculate an empirical *P*-value. Next, for the same set of 100 SNPs, we compared between the sexes the direction of effect by implementing a binomial sign test. We compared the proportion of SNPs associated in the same direction in the true results to the sex-permuted datasets to calculate an empirical *P*-value.

### Defining Biological Sets of Interests for Hormonal or Pleiotropic Mechanisms

We defined several autosomal gene sets of interest, including a 5kb flanking region when defining each gene. For all five gene sets, we removed genes based on the following criteria (1) duplicated gene name listed, (2) no corresponding SNPs in the genotype dataset, (3) gene length greater than 92Mb for appropriate length-matching, and/or (4) on the X or Y chromosome.

Androgen-responsive (AR) gene list was gathered from Androgen Responsive Gene Database (ARGDB, see URLs) for a total of 2,613 genes[55]. Of these 2,613 genes, 2,070 genes met our criteria. Estrogen-responsive (ER) gene list was gathered from Estrogen Responsive Gene Database (ERGDB), with a total of 1,384 genes[56], of which 1,092 genes met our criteria. Sexually-dimorphic (SD) genes were defined as those previously shown to have sex-biased expression patterns in the fetal brain, for a total of 285 genes, of which 227 genes met our criteria[57].

Sex-correlated (SC) genes were defined based on a number of fetal brain gene expression datasets: 1) ABI.RNAseq.21.to.26: RNAseq data from a variety of cortical areas and individuals aged 21 to 26 post-conception weeks (PCW); 2) Sestan.STHB.19.to.37: Affy Exon data from a variety of cortical areas and individuals aged 19 to 37 PCW[57]; 3) ABI.4CTX.Cingulate: Agilent arrays analyzing laser micro-dissected samples spanning the entire developing wall of cingulate cortex from four individuals (15–22 PCW); 4) STHB.STR.8.to.22: Affy Exon data from ventral telencephalon, individuals aged 8 to 22 PCW[57]; 5) AFFYEXON.4to6.mo: Affy Exon data from various brain regions in 4 to 6 month individuals[57]. [Note that these datasets may not be entirely independent of each other.] For each dataset, we found the coexpression module most significantly enriched with a set of genes previously found to be differentially expressed between males and females in human cerebral cortex[85]. These modules were summarized by their first principal component and all genes (or probes) in each dataset were correlated to PC1. These correlations were Fisher-transformed and averaged across datasets with weights corresponding to sample sizes. These values were then converted into 'average' correlation coefficients (r) using the reverse Fisher transformation and ranked genome-wide. Y-chromosome genes dominate the signature, thus genes were considered male-correlated with a Pearson correlation coefficient r > 0.3 and male anti-correlated with r < -0.3 to include moderate and strong association. Based on our criteria, we found a total of 826 autosomal male correlated genes and 58 autosomal male anti-correlated genes with corresponding SNPs in the imputed genotype dataset. For each gene set (AR, ER, SD, and SC), SNPs falling within +/- 5 kb of each gene were extracted for analysis and filtered to contain no duplicate SNPs.

Anthropometric-heterogeneous (AH) SNPs were defined in the GIANT datasets [body mass index (BMI), hip circumference (HIP), HIP adjusted for BMI (HIPadjBMI), waist circumference (WC), WC adjusted for BMI (WCadjBMI), waist-to-hip-ratio (WHR), WHR adjusted for BMI (WHRadjBMI), height, and weight][58] as showing sex difference in association with any anthropometric trait (*P* < 10^−3^). The SNP list was filtered to contain no duplicates and was LD-pruned for a final total of 8,140 SNPs, of which 3,238 overlap with the imputed ASD genotype dataset.

### Gene Set Enrichment Analysis by Permutation

In order to assess significance of enrichment in the AR, ER, SD, and SC gene sets of interest, 100 permuted gene sets with individually length-matched genes were chosen to match the true gene sets. Gene and size information were downloaded from RefSeq database—UCSC genome browser (see URLs). For each gene in the set, a gene was randomly selected from the 100 genes most similar in length to the gene of interest. For permuted gene sets, SNPs falling within +/- 5 kb of each length-matched gene were extracted for analysis. *CCSER*, *CNTNAP2*, *CSMD3*, *CTNNA2*, *DPP6*, *GRID2*, *LRP1B*, and *MACROD2* genes were too large to be matched for permutation and therefore were excluded from all gene set investigation. In order to assess significance of enrichment for the AH SNPs, 100 permuted lists of SNPs equally associated with the anthropometric traits for which the AH SNPs show sexual dimorphism were generated. For each AH SNP of interest, a SNP was randomly selected for the permuted list from 100 SNPs with the most similar trait association *P*-value. Association signal in the true biological sets of interest were compared to the permuted lists to derive an empirical *P*-value. We used the consistent FDR q-value = 0.8 threshold to determine association enrichment (see above).

In addition, we tested for binding site enrichment of AH SNPs compared to permuted lists of SNPs equally associated with anthropometric traits but not ascertained for sexual-dimorphism. The hormone-responsive transcription factors (TF) we tested included: estrogen receptor 1 (*ESR1*), estrogen receptor 2 (*ESR2*), estrogen-related receptor alpha (*ESRRA*), estrogen-related receptor beta (*ESRRB*), NK3 homeobox 1 (*NKX3-1*), lymphoid enhancer-binding factor 1 (*LEF1*), and androgen receptor (*AR*). UCSC Hg19 Table Browser[86] was used to get the 50bp upstream and downstream DNA sequence surrounding each SNP. These sequences for the AH SNPs and for the permuted SNPs lists were used as input into Deepbind[63] which used deep learning techniques to predict the binding of the hormone-responsive TF to the specified sequences. For each TF, we compared the number of sequences in permuted lists with binding scores above a threshold corresponding to the top hundredth sequence in the true AH sequences to reach an empirical *P*-value for the TF binding site enrichment.

### Power Analysis and Multiple Testing

Power analysis conducted prior to analysis suggested that for our study goal of 2,000 affected individuals in the female-only (smallest) dataset for a trio design, we would have approximately 80% power at *P*-value 5 x 10^−8^ to detect a genotype relative risk of at least 1.35 for common alleles (MAF 30%). This effect size was in the range of reported effects for other GWAS studies at the time, particularly considering that our hypothesis was that the lower-prevalence sex might contain stronger risk alleles. This analysis is simplistic, considering that our study design of meta-analyzing a small case-control cohort with the larger trio dataset is not accounted for [per affected individual, power is increased for our case-control subset– 0.78X cases are required for equal power]. Further, the power calculation was performed in order to assess the adequacy of our sample size and thus considers only a single genome-wide association analysis and none of the other kinds of analyses we performed and tested empirically.

We determined that power was insufficient for direct comparison of heritability between male and female ASD-affected probands, since for a 10% difference in heritability, we would have only approximately 30% power[87]. As risk scores can be analyzed much like any quantitative trait, our power for a t-test was adequate to detect large case vs. control differences (80% power for 0.13 SD). However, power was limited to detect more subtle potential male vs. female differences (magnitude of mean difference would need to be 67% of that observed for male case vs. control mean difference to achieve 80% power; empirical sex difference in means was 16% of the case-control difference).

Although each individual analysis is adequately corrected for multiple testing either by significance threshold or permutation, we have not accounted for the three datasets utilized (male, female, all), the five major hypotheses we are testing, nor the multiple approaches used to assess evidence for each hypothesis. Therefore, our results should be interpreted in light of the limitations of our multi-faceted study design.

### Accessions

The accession number for the UCSF ASD genotype data reported in this paper is The National Database for Autism Research (NDAR) ID 1883.

### R scripts are available in GitHub repository

**Sex permutation datasets.**https://github.com/michelaTra/ASD_SS_Mitra_I_2016/blob/master/sex_permutation_CC_trios_creator.R

**Spiked datasets.**https://github.com/michelaTra/ASD_SS_Mitra_I_2016/blob/master/risk_score_spike_set_creator.R

**Sign test**. https://github.com/michelaTra/ASD_SS_Mitra_I_2016/blob/master/sign_test.R

**Additional R functions and utilities**. https://github.com/michelaTra/ASD_SS_Mitra_I_2016/blob/master/pipeline_function.R

https://github.com/michelaTra/ASD_SS_Mitra_I_2016/blob/master/utils.R

https://github.com/michelaTra/ASD_SS_Mitra_I_2016/blob/master/pulling_variant_windows_function.R

### URLs/ Web Resources

1000G phase1v3 reference data: https://mathgen.stats.ox.ac.uk/impute/data_download_1000G_phase1_integrated.html

Androgen Responsive Gene Database (ARGDB): http://argdb.fudan.edu.cn/

Autism Genetic Resource Exchange (AGRE): http://agre.autismspeaks.org/site/c.lwLZKnN1LtH/b.5332889/k.B473/AGRE.htm

Autism Genome Project (AGP): http://www.autismspeaks.org/science/initiatives/autism-genome-project

Autism Phenome Project (APP): http://nationalautismnetwork.com/research/research-initiatives/autism-genome-project.html

Childhood Autism Risks from Genetics and the Environment (CHARGE): http://beincharge.ucdavis.edu/

DeepBind Predictive Models: http://tools.genes.toronto.edu/deepbind/

Genome-wide Complex Trait Analysis (GCTA): http://cnsgenomics.com/software/gcta/

HapMap b37: http://www.shapeit.fr/files/genetic_map_b37.tar.gz

IMPUTE2: https://mathgen.stats.ox.ac.uk/impute/impute_v2.html

Interactive Autism Network (IAN): http://iancommunity.org/cs/ian_research/ian_genetics

LiftOver—University of California Santa Cruz (UCSC) Genome Browser: https://genome.ucsc.edu/cgi-bin/hgLiftOver

LocusZoom: http://locuszoom.sph.umich.edu/locuszoom/

METASOFT: http://genetics.cs.ucla.edu/meta

National Database for Autism Research (NDAR): https://ndar.nih.gov/

PLINK: http://pngu.mgh.harvard.edu/~purcell/plink/index.shtml

R—A language and environment for statistical computing: http://www.R-project.org/

RefSeq Genes Database–UCSC: http://hgdownload.cse.ucsc.edu/goldenPath/hg19/database/knownToRefSeq.txt.gz

Simons Simplex Collection (SSC): http://sfari.org/resources/autism-cohorts/simons-simplex-collection

Study to Explore Early Development (SEED): http://www.cdc.gov/ncbddd/autism/seed.html

UCSC Table Browser: http://genome.ucsc.edu/cgi-bin/hgText

## Supporting Information

S1 Note Additional Materials and Methods. The first section describes the methods used for diagnosis of ASD in each dataset and the second section reports the IQ and DQ data available for a subset of cohorts and the criteria applied to assign each individual to low IQ or high IQ categories.
(PDF)

S1 Table Sex heterogeneity results for top GWAS association results. Heterogeneity (Cochran's Q *P*-value) between male versus female association results for the most significant SNPs in the sex-combined, male-specific and female-specific results. Logistic regression results are shown comparing low IQ (< 70) and high IQ (> 80) groups by sex.
(DOCX)

S2 Table X chromosome association enrichment. The association enrichment of sex-permuted data at an FDR q-value threshold of 0.8 is compared to the true male-specific and female-specific results.
(DOCX)

S3 Table FDR thresholds for association signal analyses. The table shows the percent of top ASD association results at various FDR thresholds for the male-specific, female-specific, and combined-sex analyses.
(DOCX)

S4 Table FDR thresholds for heterogeneity analyses. The table shows the percent of Cochran’s Q results at various FDR thresholds for the most significant 100 independent autosomal results and most significant 20 independent X chromosome results in male-specific and female-specific analyses.
(DOCX)

S1 Fig Manhattan plots of region surrounding the most significant SNPs listed in Table 1. Plots were generated using LocusZoom[88] (see URLs). SNP position information based on hg19 reference version and LD and recombination rate data based on 1000 Genomes (November 2014) EUR population for autosomal SNPs and 1000 Genomes (March 2012) EUR population for X chromosome SNPs. SNPs are colored based on linkage disequilibrium (LD) correlation (r^2^), or colored gray if no LD information exists. The overlaid blue line corresponds to the recombination rate.
(TIF)

S2 Fig Genetic risk scores by sex and IQ group. Boxplots of genetic risk scores are shown for each within-sex IQ group overlapping with the independent test datasets (low: IQ < 70 [N_female_ = 313, N_male_ = 299]; high: IQ > 80 [N_female_ = 189, N_male_ = 235]). Female data are shown in light grey and male data in dark grey. No evidence for significant differences across the groups was observed.
(TIF)

S3 Fig Multi-dimensional scaling plot. Individuals in the combined ASD dataset are plotted on the first two principal coordinates based on genome-wide SNP data. Each individual is represented with a dot and the distance between two individuals represents the genetic distance between them.
(TIF)
