# Correction: Pleiotropic Mechanisms Indicated for Sex Differences in Autism

**DOI:** 10.1371/journal.pgen.1006831

**Published:** 2017-06-07

**Authors:** Ileena Mitra, Kathryn Tsang, Christine Ladd-Acosta, Lisa A. Croen, Kimberly A. Aldinger, Robert L. Hendren, Michela Traglia, Alinoë Lavillaureix, Noah Zaitlen, Michael C. Oldham, Pat Levitt, Stanley Nelson, David G. Amaral, Irva Hertz-Picciotto, M. Daniele Fallin, Lauren A. Weiss

The fourteenth author's name is spelled incorrectly. The correct name is: Irva Hertz-Picciotto.
