# Supplementary material for: Pleiotropic Mechanisms Indicated for Sex Differences in Autism
Source: PLoS Genet. 2016 Nov 15;12(11):e1006425. doi: 10.1371/journal.pgen.1006425 (PMC5147776; doi:10.1371/journal.pgen.1006425)
Supplement: S1 Note — The first section describes the methods used for diagnosis of ASD in each dataset and the second section reports the IQ and DQ data available for a subset of cohorts and the criteria applied to assign each individual to low IQ or high IQ categories. (PDF) [file pgen.1006425.s001.pdf]

Pleiotropic mechanisms indicated for sex differences in autism.

Supplementary Note 1

Additional Material and Methods:

Diagnosis of Autism Spectrum Disorder Datasets

University of California San Francisco (UCSF)/Weiss: Autism Spectrum Disorder (ASD) probands were recruited with a clinical diagnosis of an ASD. Individuals with known genetic cause (e.g. Rett syndrome, Fragile X) were excluded.

UCSF/Hendren[1,2]: Diagnosis of an ASD in all participants was confirmed by the Diagnostic and Statistical Manual-IV: TR criteria[3], as well as the Autism Diagnostic Observation Schedule (ADOS)[4] conducted by a licensed psychologist trained to research reliability. If any diagnostic questions arose after the above were completed, the Autism Diagnostic Interview Revised was also conducted, followed by diagnostic agreement from a consensus rater's meeting reviewing all available diagnostic information. All subjects were required to have a nonverbal IQ of 49 or above, confirmed by the Wechsler Preschool and Primary Scale of Intelligence[5], Mullen Scales of Early Learning (MSEL)[6], or the Wechsler Intelligence Scale for Children[7] conducted by a licensed psychologist.

Tummy Troubles (TT)[8,9]: To confirm ASD diagnoses, children (5-18 years old) in the ASD group were assessed with the ADOS. Exclusion criteria included severe sensory or motor impairment, neurodevelopmental disorders of known etiology (e.g. Fragile X Syndrome), gestational age less than 36 or greater than 42 weeks, and birth weight less than 2,500 grams. These individuals were also recruited on the basis of positive/negative GI symptoms as described[8].

Interactive Autism Network (IAN)[10]: The Interactive Autism Network (IAN) is the largest online registry of families with an affected child with ASD in the US, with approximately 10,000 families registered. The IAN Genetics Project was initiated in 2010 for rapid recruitment and biobanking to increase sample size in genetic studies. In order to participate, an individual must be a consented participant in IAN Research:

adults (individuals aged 18 years or older) must have consented for themselves or been consented by a legally authorized representative, children must be aged 4 years or older and under age 18 years. SCQ-lifetime questionnaires were used to support reported ASD diagnoses (SCQ score at least 12). IAN reported diagnoses were previously validated[10].

Childhood Autism Risks from Genetics and the Environment (CHARGE)[11]: Eligibility is ages 2 to 5 years at enrollment, born in California, living with biological mother, parent and child speak English or Spanish, current residence in a 22 county region of California. Exclusions: children with such severe disabilities such that standardized instruments would provide invalid measures, e.g. blindness. All children who were recruited with a previous diagnosis of autism or an ASD were evaluated using ADOS[4] and Autism Diagnostic Interview, Revised (ADI-R)[12]. Only confirmed cases, based on standard criteria were classified as ASD[13]. All control children who were recruited from the general population or from a group with developmental delays other than an ASD were administered the MSEL[6], Vineland Adaptive Behavior Scales (VABS)[14], and the Social Communication Questionnaire (SCQ)[15]. General population controls whose scores were higher than two standard deviations (SD) below the mean on one and higher than 1.5 SD below mean on the other, and <12 on the SCQ were classified as typically developed controls. Any child who scored >12 on the SCQ was further evaluated using the ADOS and ADI-R. If they met criteria for an ASD, they were classified as ASD. All tests were administered by trained clinicians who had attained research reliability on the instruments they conducted.

Autism Phenome Project (APP): Inclusion criteria for a diagnosis of an ASD were based on the NIH Collaborative Programs of Excellence in Autism network. These involved: (1) meeting either the Autism Diagnostic Observation Schedule-Generic (ADOS-G)[4] cut-off score for autistic disorder or pervasive development disorder (PDD), (2) or meeting the Autism Diagnostic Interview-Revised (ADI-R)[12] cutoff score for autistic disorder and scoring within two points of this cutoff on the other measure i.e. within 2 points on ADI-R or 2 points on ADOS, (3) combined with clinical judgment. The typically developing (TD) children were screened and included after assessment with the SCQ (excluded if scores >11) (SCQ[15] – Lifetime Edition) ruled out ASD risk and the MSEL[6] revealed developmental scores 2 SD of the mean

for performance quotient and verbal quotient subscales. Exclusion criteria for controls included a diagnosis of specific language impairment, or any known developmental, neurological, or behavioral problems. Further inclusion criteria for all children, both controls and children with ASDs, included being native English speakers, ambulatory, and with no suspected vision or hearing problems. All diagnostic assessments were conducted or directly observed by trained, licensed clinical psychologists who specialize in ASD and had been trained according to research standards for these tools.

Study to Explore Early Development (SEED)[16]: SEED is a multi-site case-control study with sites in California, Colorado, Georgia, Maryland, North Carolina, and Pennsylvania. Children born from September 1, 2003, through August 31, 2006 in a catchment area, resident there at the first study contact, having a caregiver who could communicate in English (or Spanish at two sites), and within the age range for validated study instruments were eligible. Access to birth certificates and legal consent were required. ASD ascertainment was through a broad array of sources serving or evaluating children with developmental problems or by direct parent contact to the study. Individuals with an ASD or related diagnosis (e.g. intellectual disability, developmental delay) or early intervention or special education services for an ASD or related condition were considered. ASD case status was based on the results of the ADOS-G[4] and ADI-R[12], accounting for overall developmental level. Cases met ASD criteria on both the ADOS-G and ADI-R or met ASD criteria on the ADOS-G and one of three criteria on the ADI-R (i.e., met criteria on the social domain and was within two points on the communication domain, met criteria on the communication domain and was within two points on the social domain, or met criteria on the social domain and had two points noted on the behavioral domain). Controls were ascertained from birth records and subsequently underwent a limited or comprehensive developmental evaluation but did not meet the criteria (above) for ASD. Details on the SEED ASD classification algorithm can be found in Wiggins et al. (2015)[17].

IQ and DQ data available

A subset of cohorts collected one or more IQ scale assessments (see above diagnosis description). When for a single cohort more then one IQ scale assessment is available, we considered IQ scale assessments that allowed the inclusion in the analysis of the largest sample size. We assigned each individual with available IQ data to low IQ category (IQ < 70) or high IQ category (IQ > 80) based on criteria already set by the AGP cohort using verbal, non- verbal (performance) and full-scale IQ assessments [18]. For the other cohorts, we used Mullen Scales of Early Learning (MSEL) assessments as measurements of IQ scores for UCSF/Hendren[1],[2] and for Childhood Autism Risks from Genetics and the Environment (CHARGE)(11), and Vineland Adaptive Behavior Rating Scale (VABS) assessments as measurements of developmental quotient (DQ) for all the remaining cohorts. In total, IQ data were available for 3,571 affected males (2,017 low IQ, 1,554 high IQ) and 619 affected females (405 low IQ, 214 high IQ) from a subset of cohorts and used in a logistic regression analysis. We included IQ data for 1,606 affected individuals (604 low IQ and 1,002 high IQ) from Autism Genome Project (AGP) [18], for 15 affected individuals (13 low IQ and 2 high IQ) from UCSF/Hendren cohort [1],[2], for 126 affected individuals (97 low IQ and 29 high IQ) collected by Autism Phenome Project (APP), for 384 affected individuals (290 low IQ and 94 high IQ) from Childhood Autism Risks from Genetics and the Environment (CHARGE) [11] for 1,329 individuals (751 low IQ and 578 high IQ) from SSC cohort [19] and for 730 individuals (667 low IQ and 63 high IQ) from Autism Genetic Resource Exchange (AGRE) cohort [20],[21].

Additional References:

1. Bertoglio K, Jill James S, Deprey L, Brule N, Hendren RL. Pilot study of the effect of methyl B12 treatment on behavioral and biomarker measures in children with autism. J Altern Complement Med. 2010;16: 555–60. doi:10.1089/acm.2009.0177

2. Lit L, Sharp FR, Bertoglio K, Stamova B, Ander BP, Sossong AD, et al. Gene expression in blood is associated with risperidone response in children with autism spectrum disorders. Pharmacogenomics J. 2012;12: 368–71. doi:10.1038/tpj.2011.23

3. American Psychiatric Association. DSM-IV Diagnostic and Statistical Manual of Mental Disorders. Diagnostic and Statistical Manual of Mental Disorders 4th edition TR. 2000.

4. Lord C, Risi S, Lambrecht L, Cook EH, Leventhal BL, DiLavore PC, et al. The autism diagnostic observation schedule-generic: a standard measure of social and communication deficits associated with the spectrum of autism. J Autism Dev Disord. 2000;30: 205–23.

5. Wechsler D. Wechsler Preschool and Primary Scale of Intelligence. In: D.P. Flanagan, J.L. Genshaft PLH, editor. Wechsler Preschool and Primary Scale of Intelligence. The Guilford Press; 2002. pp. 120–130. doi:10.1007/978-1-4419-1698-3_866

6. Mullen EM. Mullen Scales of Early Learning Manual. American Guidance Service; 1995.

7. Wechsler D. The Wechsler intelligence scale for children—fourth edition. The Wechsler intelligence scale for children—fourth edition. 2004.

8. Gorrindo P, Williams KC, Lee EB, Walker LS, McGrew SG, Levitt P. Gastrointestinal Dysfunction in Autism: Parental Report, Clinical Evaluation, and Associated Factors. Autism Res. 2012;5: 101–108. doi:10.1002/aur.237

9. Bone D, Lee C-C, Black MP, Williams ME, Lee S, Levitt P, et al. The psychologist as an interlocutor in autism spectrum disorder assessment: insights from a study of spontaneous prosody. J Speech Lang Hear Res. 2014;57: 1162–77. doi:10.1044/2014_JSLHR-S-13-0062

10. Lee H, Marvin AR, Watson T, Piggot J, Law JK, Law PA, et al. Accuracy of phenotyping of autistic

children based on Internet implemented parent report. Am J Med Genet B Neuropsychiatr Genet. 2010;153B: 1119–26. doi:10.1002/ajmg.b.31103

11. Hertz-Picciotto I, Croen LA, Hansen R, Jones CR, van de Water J, Pessah IN. The CHARGE study: an epidemiologic investigation of genetic and environmental factors contributing to autism. Environ Health Perspect. 2006;114: 1119–25.

12. Lord C, Rutter M, Le Couteur A. Autism Diagnostic Interview-Revised: a revised version of a diagnostic interview for caregivers of individuals with possible pervasive developmental disorders. J Autism Dev Disord. 1994;24: 659–85.

13. Risi S, Lord C, Gotham K, Corsello C, Chrysler C, Szatmari P, et al. Combining information from multiple sources in the diagnosis of autism spectrum disorders. J Am Acad Child Adolesc Psychiatry. 2006;45: 1094–103. doi:10.1097/01.chi.0000227880.42780.0e

14. Sparrow SS, Cicchetti D V., Balla DA. Vineland-II Adaptive Behavior Scales: Survey Forms Manual. Second Edi. Circle Pines, MN: AGS Publishing; 2005.

15. Rutter Bailey, A., & Lord, C. M. Social Communication Questionnaire. Los Angeles, CA. 2003;

16. Schendel DE, Diguiseppi C, Croen LA, Fallin MD, Reed PL, Schieve LA, et al. The Study to Explore Early Development (SEED): a multisite epidemiologic study of autism by the Centers for Autism and Developmental Disabilities Research and Epidemiology (CADDRE) network. J Autism Dev Disord. 2012;42: 2121–40. doi:10.1007/s10803-012-1461-8

17. Wiggins LD, Levy SE, Daniels J, Schieve L, Croen LA, DiGuiseppi C, et al. Autism Spectrum Disorder Symptoms Among Children Enrolled in the Study to Explore Early Development (SEED). J Autism Dev Disord. 2015;45: 3183–94. doi:10.1007/s10803-015-2476-8

18. Anney R, Klei L, Pinto D, Regan R, Conroy J, Magalhaes TR, et al. A genome-wide scan for common alleles affecting risk for autism. Hum Mol Genet. 2010;19: 4072–82. doi:10.1093/hmg/ddq307

19. Chaste P, Klei L, Sanders SJ, Hus V, Murtha MT, Lowe JK, et al. A Genome-wide Association Study of Autism Using the Simons Simplex Collection: Does Reducing Phenotypic Heterogeneity in Autism Increase Genetic Homogeneity? Biol Psychiatry. 2015;77: 775–784. doi:10.1016/j.biopsych.2014.09.017

20. Weiss LA, Arking DE, Daly MJ, Chakravarti A. A genome-wide linkage and association scan reveals novel loci for autism. Nature. 2009;461: 802–8. doi:10.1038/nature08490

21. Wang K, Zhang H, Ma D, Bucan M, Glessner JT, Abrahams BS, et al. Common genetic variants on 5p14.1 associate with autism spectrum disorders. Nature. 2009;459: 528–33. doi:10.1038/nature07999
