# Supplementary material for: Pleiotropic Mechanisms Indicated for Sex Differences in Autism
Source: PLoS Genet. 2016 Nov 15;12(11):e1006425. doi: 10.1371/journal.pgen.1006425 (PMC5147776; doi:10.1371/journal.pgen.1006425)
Supplement: S1 Table — Heterogeneity (Cochran's Q P-value) between male versus female association results for the most significant SNPs in the sex-combined, male-specific and female-specific results. Logistic regression results are shown comparing low IQ (< 70) and high IQ (> 80) groups by sex. (DOCX) [file pgen.1006425.s002.docx]

| **Table S1: Sex heterogeneity results for top GWAS association results.** | | | | | | | | | | | |
| --- | --- | --- | --- | --- | --- | --- | --- | --- | --- | --- | --- |
| **SNP** | **CHR** | **BP** | **ALL *P*** | **ALL β** | **M *P*** | **M β** | **F *P*** | **F β** | **Cochran's Q *P*** | **L vs H IQ**  **M *P*** | **L vs H IQ**  **F *P*** |
| rs60443693 | 2 | 81439635 | 0.44 | -0.03 | 0.099 | 0.08 | 3.0x10^-8^ | -0.58 | 9.8x10^-9^ | 0.41 | 0.90 |
| rs150388754 | 8 | 4037697 | 8.3x10^-3^ | -0.15 | 0.75 | -0.02 | 8.7x10^-7^ | -0.69 | 1.4x10^-5^ | 0.52 | 0.74 |
| rs7803848 | 7 | 133108547 | 7.7x10^-6^ | -0.11 | 0.014 | -0.06 | 2.7x10^-7^ | -0.29 | 3.0x10^-4^ | 0.32 | 0.82 |
| rs150278852 | X | 140490159 | 7.4x10^-5^ | -0.53 | 2.7x10^-7^ | -0.82 | 0.36 | 0.23 | 4.2x10^-4^ | 0.17 | 0.21 |
| rs6961764 | 7 | 133131298 | 6.1x10^-7^ | 0.11 | 1.1x10^-3^ | 0.08 | 2.4x10^-6^ | 0.25 | 3.6x10^-3^ | 0.01 | 0.73 |
| rs145339701 | X | 126205770 | 8.6x10^-6^ | -0.45 | 6.4x10^-7^ | -0.56 | 0.71 | 0.09 | 0.012 | 0.85 | 0.77 |
| rs9348610 | 6 | 23812225 | 1.0x10^-6^ | -0.12 | 1.6x10^-7^ | -0.14 | 0.83 | -0.01 | 0.055 | 0.36 | 0.86 |
| rs7836146 | 8 | 119095022 | 5.6x10^-9^ | -0.17 | 6.6x10^-9^ | -0.18 | 0.18 | -0.10 | 0.29 | 0.66 | 0.35 |
| rs144955418 | X | 141650006 | 8.1x10^-8^ | -0.56 | 4.1x10^-7^ | -0.60 | 0.052 | -0.44 | 0.54 | 0.13 | 0.72 |
| rs113648237 | X | 5359798 | 7.6x10^-7^ | -0.40 | 5.8x10^-6^ | -0.43 | 0.053 | -0.31 | 0.55 | 0.28 | 0.78 |
| rs117135939 | 19 | 53743855 | 5.6x10^-7^ | 0.24 | 1.1x10^-5^ | 0.23 | 0.024 | 0.25 | 0.87 | 0.80 | 0.27 |
