# Supplementary material for: Pleiotropic Mechanisms Indicated for Sex Differences in Autism
Source: PLoS Genet. 2016 Nov 15;12(11):e1006425. doi: 10.1371/journal.pgen.1006425 (PMC5147776; doi:10.1371/journal.pgen.1006425)
Supplement: S2 Table — The association enrichment of sex-permuted data at an FDR q-value threshold of 0.8 is compared to the true male-specific and female-specific results. (DOCX) [file pgen.1006425.s003.docx]

| **Table S2. Chromosome X, 7 and 17 sex-specific association with ASD** | | | | |
| --- | --- | --- | --- | --- |
| **Chromosome** | **Association enrichment at FDR q = 0.8** | | | |
|  | **True results** | | **Sex-permutation empirical distribution** | |
|  | **M %** | **F %** | **M p-value^$^** | **F p-value^$^** |
| **X** | 29.25 | 2.29 | 0.04 | 0.36 |
| **7** | 14.49 | 2.47 | 0.63 | 0.13 |
| **17** | 24.39 | 13.07 | 0.49 | 0.29 |
| ^$^ Empirical p-value estimation based on 100 permutations | | | | |
