# Supplementary material for: Pleiotropic Mechanisms Indicated for Sex Differences in Autism
Source: PLoS Genet. 2016 Nov 15;12(11):e1006425. doi: 10.1371/journal.pgen.1006425 (PMC5147776; doi:10.1371/journal.pgen.1006425)
Supplement: S3 Table — The table shows the percent of top ASD association results at various FDR thresholds for the male-specific, female-specific, and combined-sex analyses. (DOCX) [file pgen.1006425.s004.docx]

| **Table S3. FDR thresholds for association signal analyses** | | | |
| --- | --- | --- | --- |
| **Association results** | **Chromosomes(s)** | **FDR threshold** | **% of SNPs with q-value < FDR threshold** |
| **Male-only** | Autosomes | 0.2 | 0.00 |
|  |  | 0.5 | 0.00 |
|  |  | 0.8 | 6.99 |
|  | X | 0.2 | 0.26 |
|  |  | 0.5 | 3.26 |
|  |  | 0.8 | 29.25 |
|  | 7 | 0.2 | 0.00 |
|  |  | 0.5 | 0.15 |
|  |  | 0.8 | 14.49 |
|  | 17 | 0.2 | 0.00 |
|  |  | 0.5 | 11.02 |
|  |  | 0.8 | 24.39 |
| **Female-only** | Autosomes | 0.2 | 0.00 |
|  |  | 0.5 | 0.01 |
|  |  | 0.8 | 0.25 |
|  | X | 0.2 | 0.61 |
|  |  | 0.5 | 1.04 |
|  |  | 0.8 | 2.29 |
|  | 7 | 0.2 | 0.08 |
|  |  | 0.5 | 0.35 |
|  |  | 0.8 | 2.47 |
|  | 17 | 0.2 | 0.00 |
|  |  | 0.5 | 5.08 |
|  |  | 0.8 | 13.07 |
| **Combined sex** | Autosomes | 0.2 | 0.00 |
|  |  | 0.5 | 0.78 |
|  |  | 0.8 | 10.55 |
|  | X | 0.2 | 0.15 |
|  |  | 0.5 | 1.95 |
|  |  | 0.8 | 26.83 |
|  | 7 | 0.2 | 0.17 |
|  |  | 0.5 | 0.89 |
|  |  | 0.8 | 20.70 |
|  | 17 | 0.2 | 6.00 |
|  |  | 0.5 | 10.99 |
|  |  | 0.8 | 22.75 |
