# Supplementary material for: Pleiotropic Mechanisms Indicated for Sex Differences in Autism
Source: PLoS Genet. 2016 Nov 15;12(11):e1006425. doi: 10.1371/journal.pgen.1006425 (PMC5147776; doi:10.1371/journal.pgen.1006425)
Supplement: S4 Table — The table shows the percent of Cochran’s Q results at various FDR thresholds for the most significant 100 independent autosomal results and most significant 20 independent X chromosome results in male-specific and female-specific analyses. (DOCX) [file pgen.1006425.s005.docx]

| **Table S4. FDR thresholds for heterogeneity analyses** | | | |
| --- | --- | --- | --- |
| **Cochran’s Q results** | **Chromosome(s)** | **FDR threshold** | **% of SNPs with q-value < FDR threshold** |
| **Male-only** | Autosomes | 0.001 | 0.00 |
|  |  | 0.01 | 0.00 |
|  |  | 0.1 | 16.00 |
|  |  | 0.2 | 53.00 |
|  |  | 0.3 | 59.00 |
|  | X | 0.001 | 0.00 |
|  |  | 0.01 | 20.00 |
|  |  | 0.1 | 65.00 |
|  |  | 0.2 | 70.00 |
|  |  | 0.3 | 85.00 |
| **Female-only** | Autosomes | 0.0001 | 1.00 |
|  |  | 0.001 | 66.00 |
|  |  | 0.01 | 98.00 |
|  |  | 0.1 | 100.00 |
|  |  | 0.2 | 100.00 |
|  | X | 0.0001 | 0.00 |
|  |  | 0.001 | 0.00 |
|  |  | 0.01 | 80.00 |
|  |  | 0.1 | 100.00 |
|  |  | 0.2 | 100.00 |
