# Supplementary figures and images for: Pleiotropic Mechanisms Indicated for Sex Differences in Autism
Source: PLoS Genet. 2016 Nov 15;12(11):e1006425. doi: 10.1371/journal.pgen.1006425 (PMC5147776; doi:10.1371/journal.pgen.1006425)

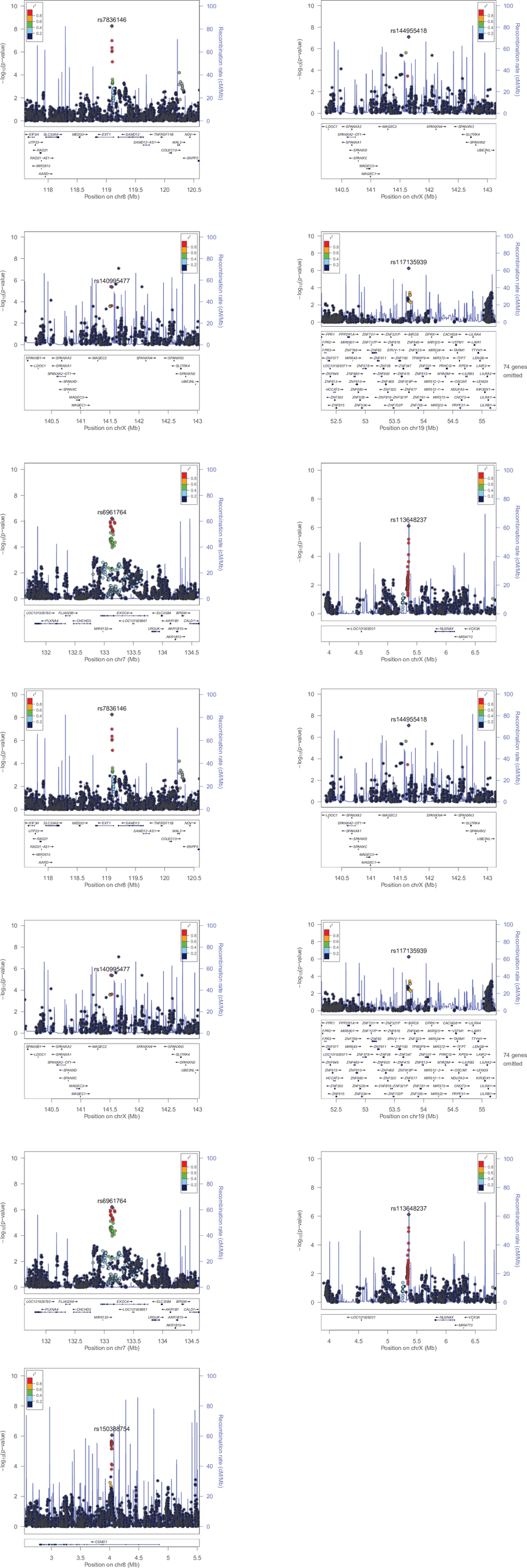

Supplement: S1 Fig — Plots were generated using LocusZoom[88] (see URLs). SNP position information based on hg19 reference version and LD and recombination rate data based on 1000 Genomes (November 2014) EUR population for autosomal SNPs and 1000 Genomes (March 2012) EUR population for X chromosome SNPs. SNPs are colored based on linkage disequilibrium (LD) correlation (r2), or colored gray if no LD information exists. The overlaid blue line corresponds to the recombination rate. (TIF) [file pgen.1006425.s006.tif]

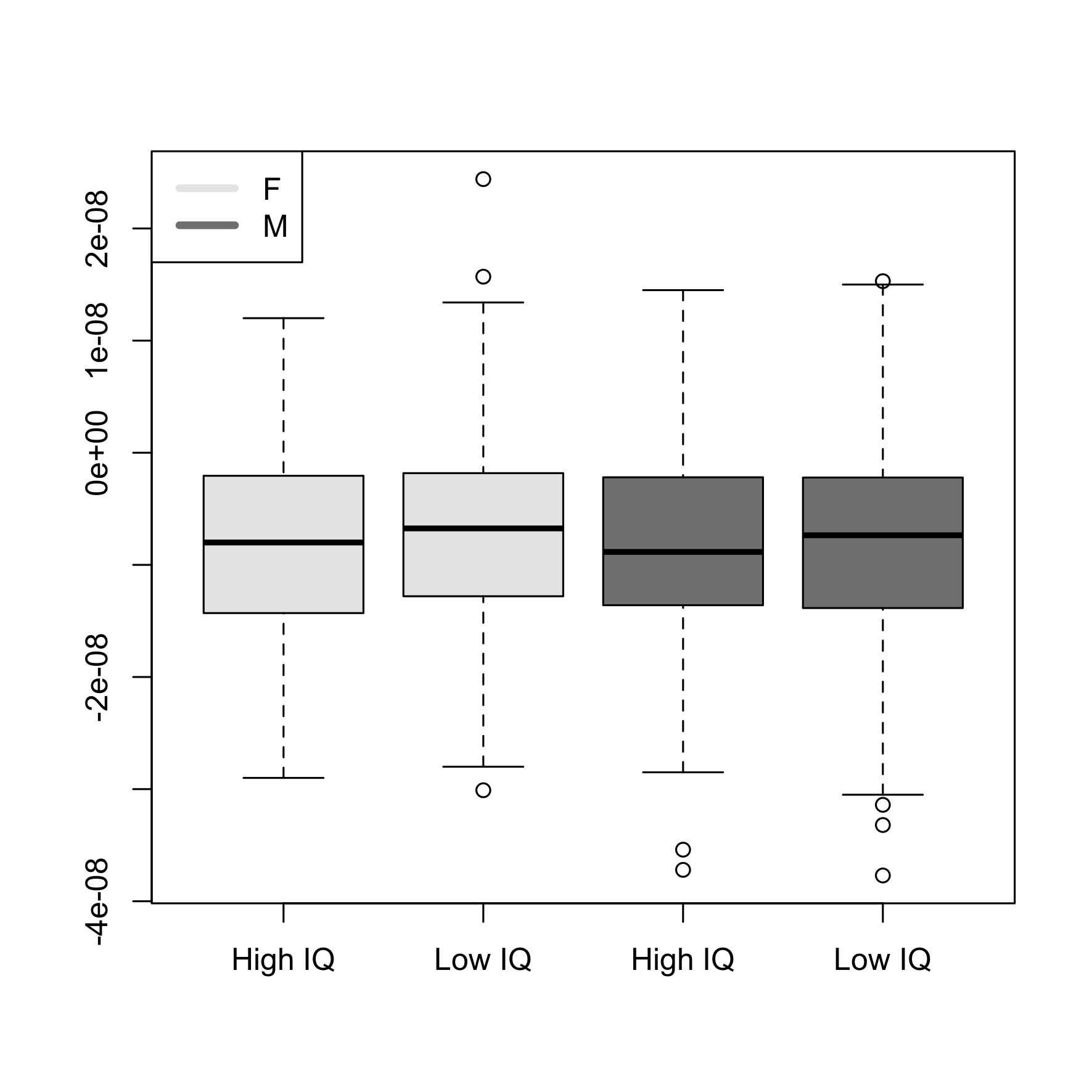

Supplement: S2 Fig — Boxplots of genetic risk scores are shown for each within-sex IQ group overlapping with the independent test datasets (low: IQ < 70 [Nfemale = 313, Nmale = 299]; high: IQ > 80 [Nfemale = 189, Nmale = 235]). Female data are shown in light grey and male data in dark grey. No evidence for significant differences across the groups was observed. (TIF) [file pgen.1006425.s007.tif]

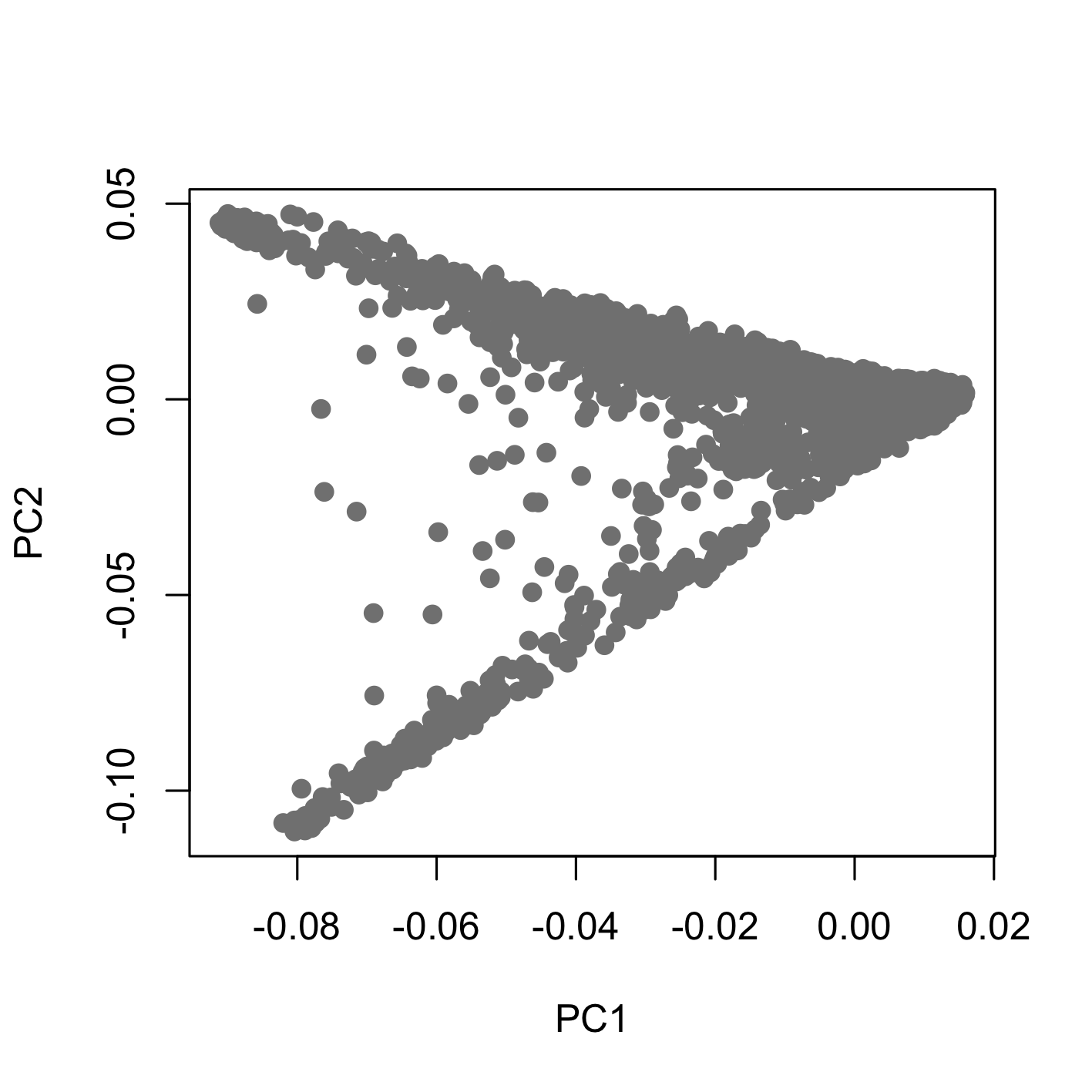

Supplement: S3 Fig — Individuals in the combined ASD dataset are plotted on the first two principal coordinates based on genome-wide SNP data. Each individual is represented with a dot and the distance between two individuals represents the genetic distance between them. (TIF) [file pgen.1006425.s008.tif]
